# Understanding of multimetallic cluster growth

**DOI:** 10.1038/ncomms10480

**Published:** 2016-01-25

**Authors:** Stefan Mitzinger, Lies Broeckaert, Werner Massa, Florian Weigend, Stefanie Dehnen

**Affiliations:** 1 Fachbereich Chemie and Wissenschaftliches Zentrum für Materialwissenschaften, Universität Marburg, Hans-Meerwein-Straße, D-35043 Marburg, Germany; 2 Institut für Nanotechnologie, Karlsruher Institut für Technologie, Hermann-von-Helmholtz Platz 1, D-76344 Eggenstein-Leopoldshafen, Germany

## Abstract

The elucidation of formation mechanisms is mandatory for understanding and planning of synthetic routes. For (bio-)organic and organometallic compounds, this has long been realized even for very complicated molecules, whereas the formation of ligand-free inorganic molecules has widely remained a black box to date. This is due to poor structural relationships between reactants and products and the lack of structurally related intermediates—due to the comparably high coordination flexibility of involved atoms. Here we report on investigations of the stepwise formation of multimetallic clusters, based on a series of crystal structures and complementary quantum-chemical studies of (Ge_2_As_2_)^2−^, (Ge_7_As_2_)^2−^, [Ta@Ge_6_As_4_]^3−^, [Ta@Ge_8_As_4_]^3−^ and [Ta@Ge_8_As_6_]^3−^. The study makes use of efficient quantum-chemical tools, enabling the first detailed screening of the energy hypersurface along the formation of ligand-free inorganic species for a semi-quantitative picture. The results can be generalized for an entire family of multimetallic clusters.

## Abstract (web summary)

Elucidation of formation mechanisms of inorganic cluster compounds is challenging due to the high coordination flexibility of the atoms involved. Here, the authors combine crystallographic and quantum-chemical studies to probe the energy hypersurface of a series of multimetallic clusters.

## Graphical abstract

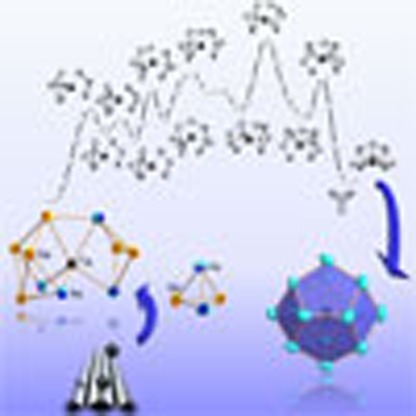

The evaluation of reaction mechanisms is not only useful but also essential for understanding, planning and optimizing chemical reactions in a reasonable and also efficient and economical way. In organic chemistry, this is a highly common procedure that allowed for the development of the retro-synthetic approach for systematic access of complex target molecules from simpler precursor fragments in the 1980s (ref. 1). However, neither this procedure nor any kind of systematic mechanistic study has so far been applied to the formation of inorganic molecules. Especially for polynuclear complexes or clusters, the formation mechanisms have widely remained unexplored to date. This is usually due to a poor structural relationship between reactants and product molecules. Furthermore, the flexibility of the metal atoms within a cluster regarding coordination numbers and geometry allows for a relatively quick re-organization, which usually prohibits monitoring of the processes and the detection of structurally related intermediates—at least in homoatomic cases, which lack any kind of ‘tracer' atom.

Metal clusters in general have been subject of countless studies over the past decades, the more as monodisperse species came into sight as veritable and controllable quantum dots^2^,^3^, which have also been used to generate novel nanostructured solids^4^,^5^,^6^. In particular, multimetallic clusters have attracted much attention by chemists and physicists in the recent past as they represent monodisperse intermetallic particles with superatom characteristics^7^ and fine-tunable opto-eletronic as well as magnetic properties^8^,^9^,^10^. For this, these clusters can be viewed as molecular models to doped metals or bimetallic, catalytically active nanostructures^11^,^12^.

However, the design of such clusters has appeared to be very challenging, since for reactions in condensed matter, the very first steps in the formation starting out from atomic, molecular or solid state precursors have essentially remained unexplored so far. It would thus be of great benefit to elucidate these processes to make such particles more generally available. A profound knowledge of all evolutionary steps would allow for overcoming the challenges of a reproducible synthesis, controlling the shape and size as well as the fine-tuning of chemical and physical properties.

A recently active investigated class of corresponding multimetallic compounds are intermetalloid clusters, that is, main-group (semi-)metal cages embedding transition metal atoms^13^,^14^,^15^,^16^. A large variety of different cluster structures has been presented over the last two decades, with ever larger and more complex architectures, that are usually obtained in solution by reaction of homoatomic or heteroatomic Zintl anions with transition metal complexes. Here, in some cases, intermediate complexes have been isolated that allowed for some understanding of the stepwise release of organic ligands from the used transition metal complexes^17^,^18^,^19^,^20^,^21^, but it was not possible to trace back the complicated re-arrangement processes of the smaller Zintl anions in the presence of transition metal atoms.

The lack of knowledge regarding the relationship between reactants and products becomes particularly obvious for non-deltahedral cluster architectures including group 14 metal atoms^11^,^22^,^23^,^24^,^25^,^26^,^27^, since all known precursors have been deltahedral main-group element polyanions so far.

Herein, we report on the synthesis and isolation of a series of compounds containing heterometallic or intermetalloid polyanions of different sizes, (Ge_2_As_2_)^2–^, (Ge_7_As_2_)^2–^, [Ta@Ge_8_As_4_]^3–^ and [Ta@Ge_8_As_6_]^3–^. During these studies, we additionally crystallized an unprecedented intermediate, [Ta@Ge_6_As_4_]^3–^, of the corresponding intermetalloid cluster anions. This result prompted us to investigate the reaction by a systematic exploration of the energy hypersurface with recently developed tools for the search of low-lying minima in mixed-metallic systems^28^ and for the optimization of transition pathways^29^. This way, we shed light on the complex formation processes behind non-deltahedral multimetallic clusters in a semi-quantitative manner.

## Results

### Experimental findings

The study started out with the synthesis of a precursor compound with a binary anion, [K([2.2.2]crypt)]_2_(Ge_2_As_2_)·*en* (**1**), which was carried out according to the syntheses of homologous or isoelectronic compounds^30^,^31^,^32^,^33^,^34^, by fusion of K, Ge and As (1:1:1) in Ta tubes at high temperatures, slow cooling to room temperature and subsequent extraction with *en*/[2.2.2]crypt at room temperature. The only difference from previous procedures was the application of a somewhat higher maximum temperature, 950 °C instead of 600 °C at the fusion step, which we initially chose in regard of the higher melting temperature of germanium as compared with those of tin or lead, and which turned out to be necessary to gain Ta atoms from the bulk (see below). As in many other cases before, we also crystallized a compound with a 9-atom binary polyanion from the extraction solution, [K([2.2.2]crypt)]_2_(Ge_7_As_2_) (**2**), and we detected a known homoatomic 10-atom cage (Ge_10_)^2–^ (**A**)^35^, along with **1** (compound **2** was also gained starting from a K/Ge/As phase generated in silica ampoules, indicating that Ta is not needed for its synthesis). However, the procedure also afforded two further compounds, which comprise ternary intermetalloid clusters with Ta atoms inside, [K([2.2.2]crypt)]_3_[Ta@Ge_6_As_4_]·2*tol* (**3**) and [K([2.2.2]crypt)]_5_[K([2.2.2]crypt)(*en*)][Ta@Ge_8_As_4_]_1.21_[Ta@Ge_8_As_6_]_0.79_*·en* (**4**).

Obviously, the higher temperature at the beginning of the synthesis and the particular elemental combination enabled the reaction with the tube material and subsequent incorporation of Ta^5+^ (as confirmed by X-ray absorption spectroscopy (EDX) analyses of the resulting solid). The highly polarizing nature of the hard Ta^5+^ ion, which increases the covalent character of the bonds between transition metal and main-group (semi-) metal atoms, is made responsible in turn for the possibility to isolate fragmentary/intermediate complexes such as the anion in **3**. According structures are not likely to be isolated in the presence of less-polarizing cations, such as the Ln^3+^ series used before.

We thereupon developed a systematic access to these phases by addition of Ta powder. All four compounds were isolated from the extraction solution in single-crystalline form, and experimentally characterized by energy dispersive EDX (Supplementary Fig. 1, Supplementary Tables 1 and 2), X-ray diffraction (Fig. 1, Supplementary Figs 2–13, Supplementary Tables 3–7) and electrospray ionization mass spectrometry (ESI-MS, Supplementary Figs 14–26). The elucidation of single-crystal structures was not trivial here as it faced the following complications: (I) indistinguishability of Ge and As atoms by MoKα radiation, inhibiting the experimental assignment of Ge/As atomic positions, (II) positional disorder of the anions (**2** and **4**) and (III) co-crystallization of diverse anions of different structures (**3**) or composition (**4**), in the latter case arising along with complication (II).

### From crystal structures to a sketched formation mechanism

**Figure 1:**
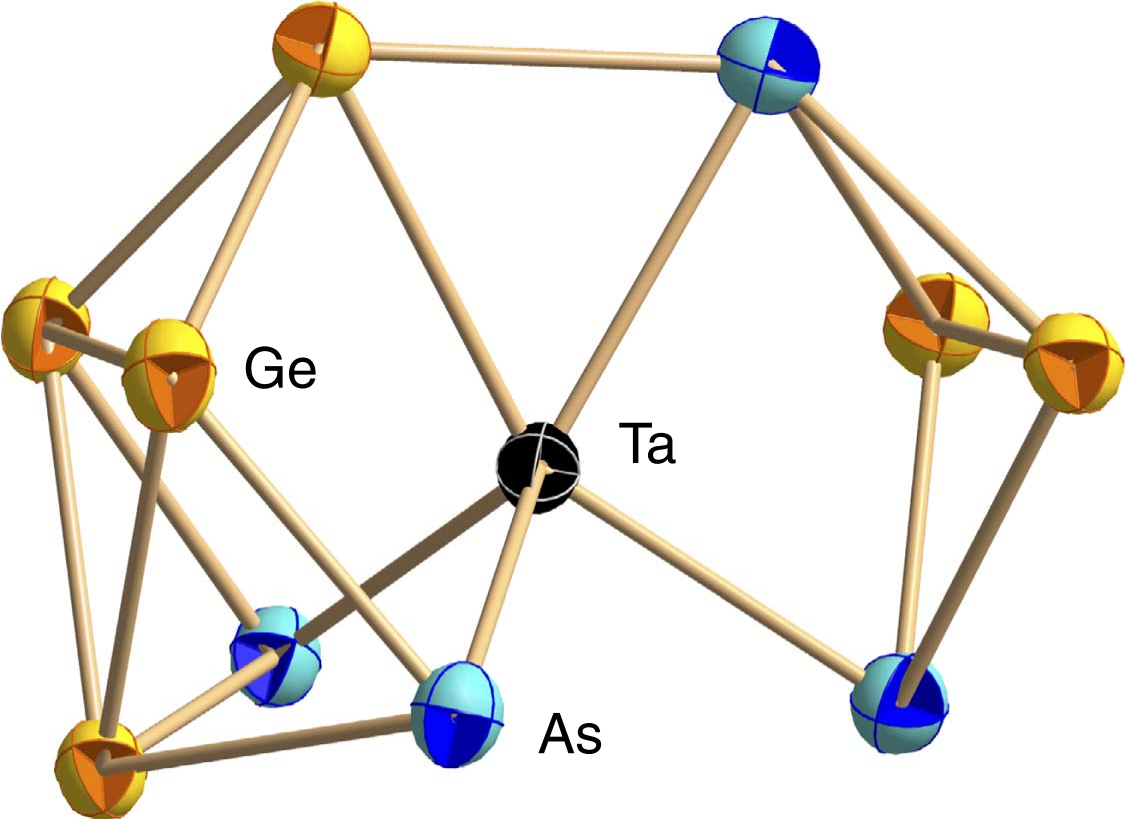
Molecular structure of the predominant anionic component in 3. Thermal ellipsoids are shown at 50% probability. Selected distances [Å]: Ge/As–Ge/As 2.4770(7)-2.761(1); Ta–Ge/As 2.496(1)-2.719(1). Owing to non-distinguishability of Ge and As atoms for X-rays, the refinement was done based on the assignment of Ga or As atoms resulting from DFT calculations. The minority component isomer is shown in Supplementary Fig. 7b.

**Figure 2:**
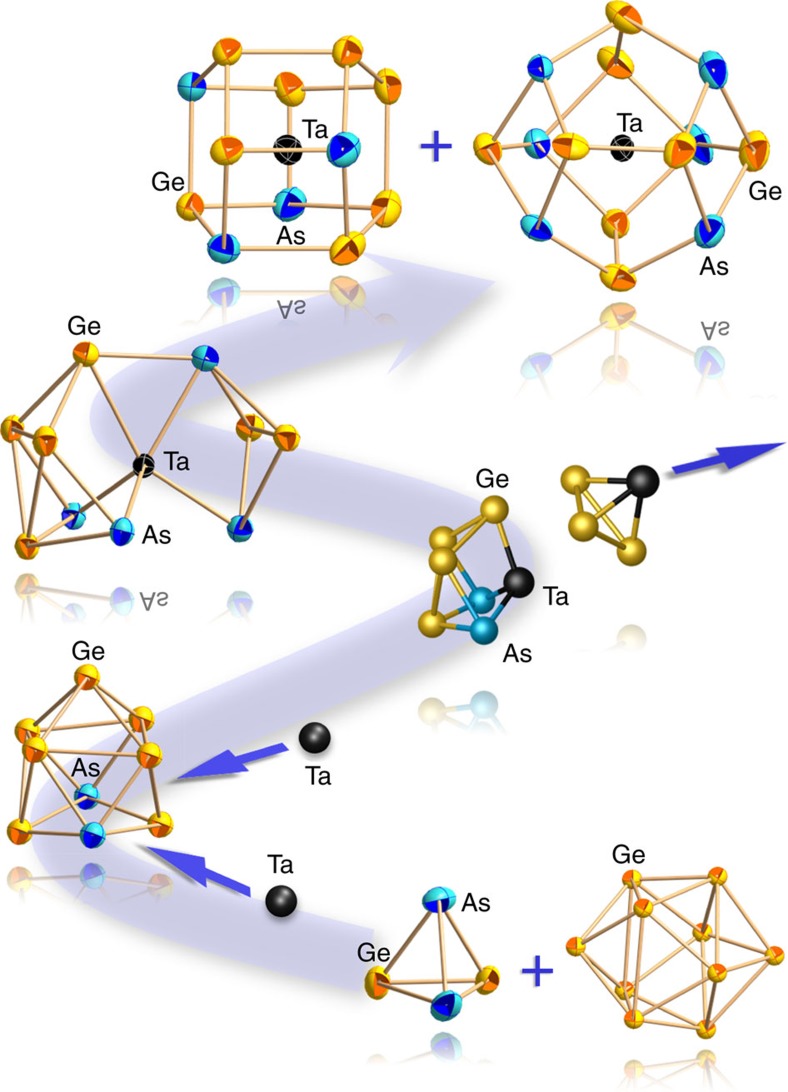
Outline of the stepwise formation of non-deltahedral intermetalloid Ta/Ge/As clusters. The shown pathway starts out from the (Ge_2_As_2_)^2−^ anions in **1** and the (Ge_10_)^2−^ anion^40^ (bottom) under consideration of all isolable species, hence the anions in **2** (2nd from bottom), in **3** (2nd from top) and in **4** (top), plus the calculated, hypothetic anions [Ta@Ge_4_As_2_]^−^ and (TaGe_3_)^−^ (centre). The minimum structures of the latter are shown in a different style of representation for easier tracking.

Besides [K([2.2.2]crypt]^+^ cations, the compounds comprise a tetrahedral (Ge_2_As_2_)^2–^ anion (**1**), a nine-atom anion (Ge_7_As_2_)^2–^ (**2**) with the topology of the well-known (Ge_9_)^4–^ cage^13^, two different isomers of [Ta@Ge_6_As_4_]^3–^ with so far unprecedented 10-atom architectures (**3**), as well as the clusters [Ta@Ge_8_As_4_]^3–^ and [Ta@Ge_8_As_6_]^3–^ (**4**). One of the co-crystallizing anions in **4** is based on a 12-atom cage with a rare non-deltahedral topology, reported recently by Goicoechea and co-workers for the binary cluster [Ru@Ge_12_]^3–^ (ref. 36), and for [V@Ge_8_As_4_]^3–^ produced in our lab^37^. The Ge/As shell of the second anion in **4** adopts the stable 14-atom enneahedron of main-group metals, which was observed recently for several lanthanide-centered examples, [Ln@Sn_x_Bi_14–x_]^4–^ (Ln/x=Eu/6; La/7, Ce/7)^25^,^26^. While the topology of the latter is known, the corresponding cluster in **4** comprises the smallest 14-atom cage known to date (Ø 5.7…5.8 Å), and it is the second intermetalloid cluster of this type that does not embed a lanthanide cation but a group 5 metal ion besides [Nb@Ge_8_As_6_]^3–^ (ref. 37). Hence, whereas the topologies of the anions in **1**, **2** and **4** are known for other elemental compositions, neither the composition nor the structures of the anions in **3** have been known so far. Figure 1 shows the molecular structure of the predominant component (90%) of the two isomeric anions in **3** (for the minority component isomer, see Supplementary Fig. 7b). The crystal structures of all other anions are presented in Fig. 2 and in Supplementary Figs 3,5,10 and 11. Full crystallographic details can be gained from Supplementary Table 3 and Supplementary Data 1–4.

According to these results, the uncommon cluster structure can be described as being composed of two parts, a 6-atom (Ge_4_As_2_) unit and a 4-atom (Ge_2_As_2_) unit, attached to a Ta atom. As will be discussed in the following, we claim this anion to be the key species for the formation of non-deltahedral, multimetallic clusters. This leads us to the following general formation protocol (equation (1)) including all observed anionic compositions, as well as Ta metal (represented by atoms here):


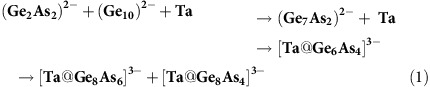


to use the information gained so far for understanding of the formation mechanism along the steps indicated in the above equation, we systematically investigated the energy hypersurface of all of the given anions. This was realized with the programme system Turbomole^38^ by a genetic algorithm (GA)^39^,^40^,^41^,^42^,^43^ based on density functional theory (DFT) with the functional by Becke and Perdew, BP86 (refs 44, 45), and polarized split-valence basis sets, def-SVP (refs 46-48, 47, 48), and further employing the conductor-like screening model (COSMO)^49^ with standard settings for charge compensation. Within the GA, one is faced with the problem of assigning Ge and As to the atomic positions in the energetically best way. This (re)assignment of atoms to places was done by first-order perturbation theory (RP), with the difference in nuclear charges of the two elements as perturbation parameter^50^,^51^. The resulting procedure, called GA-RP^28^ was applied to all systems with the settings given in ref. 28. The resulting local minimum structures for each isomer were subsequently re-optimized employing the functional by Tao, Perdew, Staroverov and Scuseria, TPSS (ref 52), with polarized triple zeta valence basis sets^53^ and Dirac-Hartree-Fock effective core potentials^54^, dhf-TZVP, which for main-group element clusters yields results close to that of coupled-cluster calculations^55^. We note in passing that the influence of different types of functionals is small, barrier heights with BP86 are lower by typically 3.8 kJ mol^−1^, maximal by 9.1 kJ mol^−1^; differences to the functional by Perdew, Burke and Ernzerhof (PBE)^56^ or to the hybrid functional by Tao, Perdew, Starovereov and Scuseria (TPSSh)^57^ functionals are even smaller, see Supplementary Tables 8 and 9. The global minima obtained from the GA-RP procedures possess topologies that are identical with those of the experimentally determined structures throughout, and all structural parameters agree well. For the species (Ge_2_As_2_)^2–^/(Ge_7_As_2_)^2–^/[Ta@Ge_6_As_4_]^3-^/[Ta@Ge_8_As_6_]^3–^/[Ta@Ge_8_As_4_]^3**–**^ the mean deviations amount to 0.027/0.019/0.012/0.017/0.033 Å, the maximum deviations to 0.062/0.097/0.037/0.074/0.104 Å. This indicates both the reliability of the procedure for their identification and the reliability of TPSS/dhf-TZVP for the description of the energy hypersurface at 0 K (disregarding zero-point energy). For all cases of interest, connecting pathways between isomers were calculated with an iterative method based on a local quadratic approximation of the energy hypersurface^29^. Subsequently, all minima and maxima of two of these pathways were re-optimized, which leads to a small increase of energies for barriers (by ca. 2 kJ mol^−1^). We convinced ourselves that all transitions in fact represent reaction coordinates. This was done by distorting each maximum along its imaginary mode in positive and negative direction and subsequently optimizing the distorted structures (minimum search). In all cases we verified that the two resulting structures are identical to the minimum structures right and left to the maximum. Moreover, thermal corrections from energies at 0 K to free enthalpies were calculated from partition sums within the standard harmonic oscillator approximation^58^ for each molecule in the gas phase; vibrational frequencies were used non-scaled. The resulting data are given in Supplementary Figs 27 and 28, Supplementary Tables 10 and 11 for *T*=298, 500, 700 and 900 K. For these temperatures, mean changes in barrier heights amount to +0.6/+3.8/+6.4/+10.1 kJ mol^−1^, maximum changes to +3.2/+7.7/+12.7/+18.3 kJ mol^−1^, respectively. The overall changes of the barrier heights resulting from the bare inspection of the pathway to those obtained when considering the above effects (individual optimization of minima/maxima, functional dependence and temperature dependence) thus are estimated to be smaller than 10 kJ mol^−1^ at room temperature and below 20 kJ mol^−1^ at *T*=900 K; changes for relative energies of minima are even smaller. These—rather moderate—effects thus were not considered in the following and reaction barriers were taken directly from the optimized reaction pathways.

Furthermore, the GA-RP procedure was used to determine the global minimum structures of further fragments that resulted from hypothetic withdrawal of atoms from the experimentally observed larger clusters. Two of such hypothetical fragments, [Ta@Ge_4_As_2_]^–^ and (TaGe_3_)^–^, turned out to be reasonable candidates for missing links in the reaction cascade, considered to form on interaction of (Ge_7_As_2_)^2–^ with Ta atoms. They were, however, not detected in the experimental studies, presumably owing to their (high) reactivity. Figure 2 provides an illustration of the stepwise cluster formation as suggested based on the preliminary results regarding all of the named species and their topologies.

### Deeper insight into the formation pathway

Although the relative amounts of isolated products are dependent to some extent on the K:Ge:As ratio of the reactants (besides an approximately constant amount of released Ta tube material of ca. 4%), the fusing temperature, the amount of solvent, the extraction time and the layering technique, it was not possible so far to isolate the separate species stepwise in real time. Mass spectrometry experiments indicate that the final clusters that co-crystallize in compound **4** can be already detected in the first spectrum recorded after injection of fresh extracts of the solid precursor phase. This indicates that either all of the detected species are formed side by side during cooling of the melt (the Ta-containing ones on contact of the K/Ge/As alloy with the Ta tube material or Ta powder), or that those reactions that occur in solution are rather quick. As explained and quantified below, the first steps leading from the anion in **1** through that in **2** to the first Ta-containing anion, [Ta@Ge_4_As_2_]^–^, require higher temperatures, as they finally come along with the release of Ta atoms from Ta metal. The formation of the anion in **3** can follow in the solid or in solution at room temperature, given that *en* would sufficiently stabilize the [Ta@Ge_4_As_2_]^–^ species. The last step, leading from the anion in **3** to the anions in **4**, is very likely a reaction in solution. For the processes being considerably rapid, we were definitely lucky to find a system that enabled the crystallization of reactants and diverse synthons along with the final products. Still, as more experimental information cannot be gained here due to the complicated and fast rearrangement processes within complex reaction mixtures, and owing to the lack of energetic information, a complementary quantum-chemical analysis of the formation process was necessary for a refined and at least semi-quantitative picture.

In this comprehensive study, we did not only consider all species mentioned above plus (Ge_3_As)^3–^ and (As_2_)^2–^ as additional leaving groups, but also the energetically higher isomers of the involved anions, which resulted from the GA-RP procedure; although the latter were not observed in stable crystals, they may play a role as reactive intermediates. Reaction energies were calculated for those steps of the reaction with atom- and charge-balance for educts and products, since only for these cases, the dependency of the calculated energy differences on the dielectric constant chosen in the COSMO approach is reasonably small (Supplementary Table 12). Reaction pathways were calculated with an iterative method based on a local quadratic approximation of the energy hypersurface^29^.

Here, we will focus on three general aspects: First, the initial step that leads to the formation of the ubiquitous nine-atomic anion (Ge_7_As_2_)^2–^ from the four-atomic and ten-atomic precursor anions (Ge_2_As_2_)^2–^ and (Ge_10_)^2–^. Second, the formation of the [Ta@Ge_6_As_4_]^3–^ anion in **3** from (Ge_7_As_2_)^2–^ through [Ta@Ge_4_As_2_]^–^. These steps are apparently intuitive regarding the development of the structures (Fig. 2), thus we will use this part of the mechanism mainly to indicate the role of the transition metal atom. The final step to form [Ta@Ge_8_As_4_]^3–^ and [Ta@Ge_8_As_6_]^3–^ from [Ta@Ge_6_As_4_]^3–^ lacks an according structural relationship. Hence, we explored the availability and the role of possible isomers of [Ta@Ge_6_As_4_]^3–^ in the third part of the following discussion.

We note in advance that the formation pathway that we derived from our combined experimental and extensive quantum-chemical study represents only one of several possible routes, but obviously a very plausible one, as it is based on ‘smooth' inter- or intramolecular re-arrangements; these are feasible with energies that are significantly smaller than those of uncompensated bond-breaking, like breaking an As–As bond in As_4_, which amounts to 179 kJ mol^−1^ (calculated as (*E*(As_4_)−4*E*(As))/6) at level TPSS/dhf-TZVP), or breaking a Ge–Ge bond (188 kJ mol^−1^)^59^. The presented route leads to a topological relation between all structures therein, and as it also bridges to all previously known non-deltahedral clusters (see below).

### Ta-independent first step

The co-existence of (E^14^_2_E^15^_2_)^2–^, (E^14^_10_)^2–^ and (E^14^_7_E^15^_2_)^2–^ anions in solutions of ternary K/E^14^/E^15^ phases^25^,^26^,^30^,^33^,^34^ is very obvious, but has heretofore never been explained. Thus, we inspected this step in detail, which at the same time represents the initial step in the intermetalloid cluster formation pathway shown in Fig. 2. The energy difference for the formation of two (Ge_7_As_2_)^2–^ anions from (Ge_10_)^2–^ and two (Ge_2_As_2_)^2–^ anions cannot be calculated reliably owing to missing charge balance (see above). For that, we performed the calculation with a total charge of 4– throughout, thus formally considering a partially oxidized precursor anion ‘(Ge_2_As_2_)^–^'. The latter, which actually represents the electronic configuration of the species found under ESI-MS conditions, was calculated to be a global minimum with an elongated Ge…Ge edge, according with the fact that the HOMO of (Ge_2_As_2_)^2–^ represents the Ge–Ge bond. Although we cannot exclude that the oxidation takes place later on during this step, an early oxidation right at the beginning seems to be reasonable in terms of easier approach of the anionic species.

**Figure 3:**
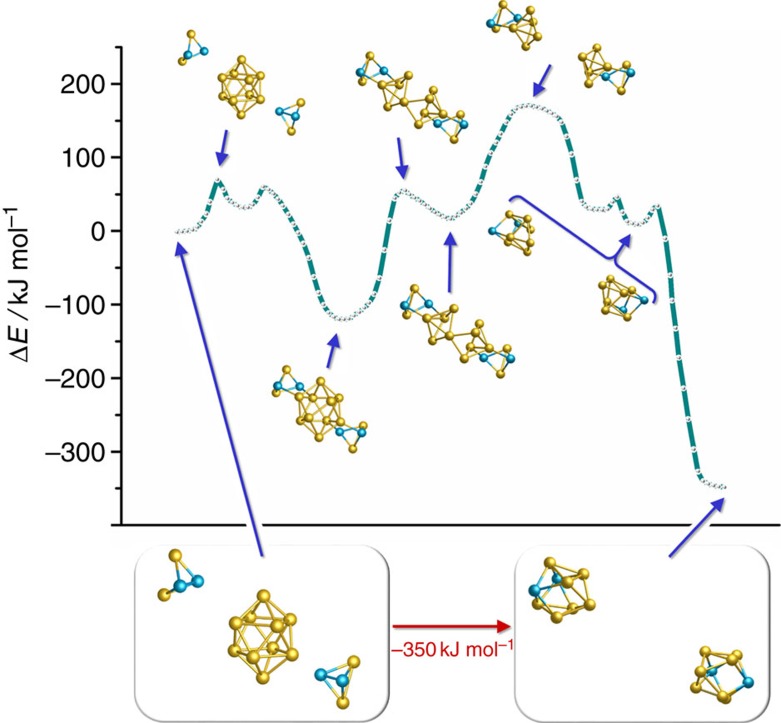
Formation of two (Ge_7_As_2_)^2−^ anions from {(Ge_2_As_2_)…(Ge_10_)…(Ge_2_As_2_)}^4−^. The pathway was modelled based on the initial arrangement of three separate anions (bottom left), the final separate (Ge_7_As_2_)^2−^ anions (bottom right) and an intermediate, local minimum structure, in which the two (Ge_2_As_2_)^−^ species are attached to the (Ge_10_)^2−^ cage in topologically reasonable way regarding the products (marked with an asterisk). Selected structures along the pathway are shown.

The reaction pathway from an initial arrangement of three separate species {(Ge_2_As_2_)…(Ge_10_)…(Ge_2_As_2_)}^4–^ to the final arrangement of two separate anions {(Ge_7_As_2_)…(Ge_7_As_2_)}^4–^ (Fig. 3) was calculated^29^ using 53 intermediate structures at level TPSS/dhf-TZVP (refs 52, 53, 54), including one intermediate optimized local minimum structure, in which the two (Ge_2_As_2_) species are attached to the (Ge_10_) cage. An asterisk in Fig. 3 marks this intermediate. The chosen type of attachment is the one that requires minimum re-arrangement for the (Ge_7_As_2_)^2–^ products (that is, further types of attachment cannot be excluded, but would cause higher barriers). The reaction pathway is summarized as follows. The intermediate is more stable than the initial arrangement by ca. 120 kJ mol^−1^, and achievable by a small barrier of ca. 70 kJ mol^−1^ (and a second, smaller one). The final arrangement in turn is more stable than the intermediate by ca. 240 kJ mol^−1^, but separated from the latter by two barriers, amounting to 180 kJ mol^−1^ and 160 kJ mol^−1^, respectively (besides some further, much smaller ones). The first of the two barriers comes along with a significant break-up of the central Ge_10_ cage. The reason for a still rather small barrier in spite of the loss of a total of five Ge–Ge contacts can be explained by changes in nature and quality of the Ge–Ge bonds. The marked isomer exhibits electron-deficient multi-centre bonding, as reflected by an average coordination number of 4.8 for the 10 Ge atoms in the central part, whereas for the higher-energy state these atoms are in a nearly electron-precise situation with an average coordination number of 3.8 (including the exo-Ge–As bonds for both cases). Thus, the decreasing number of bonds is accompanied by an increasing bond order for the remaining bonds. The second barrier mainly reflects the cleavage of the remaining Ge–Ge bond between the two newly formed fragments. The dissociation energy is in the range of a Ge–Ge single bond (188 kJ mol^−1^) (ref. 59). These newly formed fragments show a rather low-average coordination number (3.3), which increases to 3.6 as two pairs of Ge atoms approach each other (second image from right in Fig. 3) and finally to 4.0 in the nine-atom cages; the As atoms are three-bonded throughout the path before they turn into four-bonded ones during the final process. The entire reaction is exothermal by ca. 350 kJ mol^−1^, and the barriers are conquerable in the high-temperature flux.

### Inclusion of Ta and formation of the key anion in 3

Due to its shape, the [Ta@Ge_6_As_4_]^3–^ anion in **3** is most reasonably described as [Ta@Ge_4_As_2_]^–^·(Ge_2_As_2_)^2–^. This may be viewed as the result of an insertion of the [Ta@Ge_4_As_2_]^–^ part of the anion in **3** into the As–As bond (that is, the LUMO) of the original (Ge_2_As_2_)^2–^ tetrahedron. During this step, Ta is thus formally oxidized from a +3 to a +5 state. The fusion of these two parts to the anion in **3** is exothermic by 331 kJ mol^−1^ (Supplementary Table 12). The formation of the preceding species, [Ta@Ge_4_As_2_]^–^, is intuitively considered as the reaction of Ta atoms with (Ge_7_As_2_)^2–^ during the high-temperature reaction, as the former can be derived from the latter by replacement of a (Ge_3_)^2–^ ring with Ta; the (Ge_3_)^2–^ unit is thereby trapped by a second Ta atom to form (TaGe_3_)^–^ under 1-e^–^ oxidation; this species was not experimentally isolated; it should be reactive enough to immediately react with half an equivalent of (As_2_)^2–^, released in the formation of [Ta@Ge_8_As_4_]^3–^ (see final step below) to form the experimentally proven (Ge_3_As)^3–^ anion (Supplementary Fig. 19). The formation of [Ta@Ge_4_As_2_]^–^ and (TaGe_3_)^–^ from (Ge_7_As_2_)^2–^ and two Ta atoms is exothermic by 903 kJ mol^−1^ (Supplementary Table 12). Of course, the Ta atoms need to be liberated previously from the bulk phase, that is, the wall of the reaction tube. The enthalpy of atomization of Ta metal amounts to 782 kJ mol^−1^ per atom^59^, which may be the upper limit. However, for a surface atom, this energy is much smaller, in particular when considering defects like edges or corners. It should be significantly smaller than the dissociation energy of (formally) quadruple-bonded Ta_2_, 504 kJ mol^−1^ (energy difference to separated atoms at TPSS/dhf-TZVP level), so that the overall reaction will be exothermic.

If the subsequent formation of **3** takes place in solution, that is, during or shortly on the extraction step, a chelating solvent—such as *en*—is absolutely essential to intermediately stabilize the quoted interim species like [Ta@Ge_4_As_2_]^–^ or (TaGe_3_)^–^ by coordinating to the valence-unsaturated Ta atom; according *en* complexes are presumably not stable enough for detection (at least under the conditions that we applied in our measurements), but indeed, other solvents than *en* did not lead to any identifiable products in this study. Alternatively, all initial steps might already occur in the flux, such that the anions in **1–3** co-exist in the solid prior to extraction.

Independent from the very reaction conditions, the described process involves species with stepwise increasing formal oxidation state at the Ta atoms (0, +3 and +5), which is in line with the idea of successive redox cascades involving the transition metal atom.

### Isomerization processes and final step

The formation of the two clusters in **4**, [Ta@Ge_8_As_4_]^3–^ and [Ta@Ge_8_As_6_]^3–^, is not possible starting from [Ta@Ge_6_As_4_]^3–^ in its experimentally observed shape (see Fig. 1) as neither the topology nor the Ge/As distribution match. Instead, higher-energy isomers of [Ta@Ge_6_As_4_]^3–^ with more suitable topologies and Ge/As distributions with regard to the larger clusters should be the reactive ones in subsequent reactions with (Ge_2_As_2_)^2–^ (to obtain the minimum structure of [Ta@Ge_8_As_4_]^3–^ and (As_2_)^2–^ or the minimum structure of [Ta@Ge_8_As_6_]^3–^ and 2e^–^). In principle, these isomers might form as kinetic products directly from the precursors (Ge_2_As_2_)^2–^ and (Ge_10_)^2–^, or the (Ge_7_As_2_)^2–^ nine-atom cage in an alternative way than shown here; however, as this hypothesis requires much more assumptions and is not based on further experimental proof, we rather suggest a re-arrangement starting out from the experimentally observed global minimum structure of [Ta@Ge_6_As_4_]^3–^.

**Figure 4:**
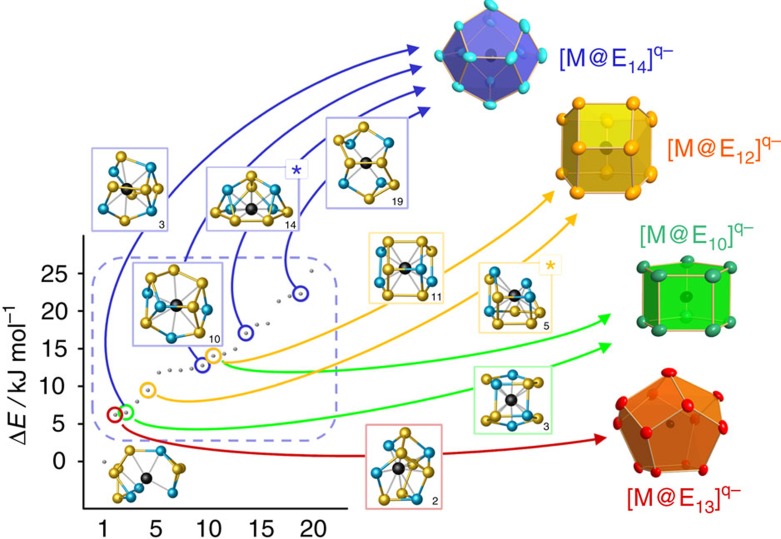
Illustration of the low-energy re-arrangement of the anion in 3 to form isomers. In addition to the global minimum structure, the energies (dashed blue box) and selected structures of the 19 isomers following in energy are given. The drawn isomers represent exact fragments of the known non-deltahedral intermetalloid cluster topologies (for references see text). Two isomers are marked with an asterisk, symbolizing those that are discussed as direct precursors to the cluster with 12 atoms (isomer 5, yellow) or 14 atoms (isomer 14, blue), respectively.

For this purpose, we inspected the lowest 20 isomers resulting from the GA-RP procedure. These isomers are higher than the minimum by ca. 5–25 kJ mol^−1^, thus their temporary existence is plausible. Many of them are fragments of regular [M@E_n_] anionic polyhedra (*n*=10–14), that is, they have topologies fitting to all experimentally known non-deltahedral intermetalloid clusters containing tetrel atoms, as illustrated in Fig. 4. This finding extends the meaningfulness of this work to a more general understanding of multimetallic cluster formation.

As mentioned above, for a subsequent reaction to [Ta@Ge_8_As_4_]^3–^ or to [Ta@Ge_8_As_6_]^3–^, additionally a matching Ge/As distribution is required. This is the case, for the fifth-stable isomer of [Ta@Ge_6_As_4_]^3–^ (isomer 5; +9 kJ mol^−1^; marked with a yellow asterisk in Fig. 4), which is the lowest energy isomer representing an exact fragment of the experimentally found topology and global minimum Ge/As distribution of [Ta@Ge_8_As_4_]^3–^. The latter (and additionally the reactive intermediate (As_2_)^2–^, see above) is formed from this fragment with (Ge_2_As_2_)^2–^ in a near isoenergetic step (–1 kJ mol^−1^). Similarly, the fourteenth-stable isomer (isomer 14; +17 kJ mol^−1^; marked with a blue asterisk in Fig. 4) is identical to the upper part of the most favourable [Ta@Ge_8_As_6_]^3–^ structure. Hence, for completion of this cluster, only a (Ge_2_As_2_) unit is lacking; since the latter is originally provided in its dianionic form, an oxidation has to take place. As this reaction thus yields different charges for educts and products, the corresponding energy could not be reliably calculated (see above). However, similar energetics can be expected as for the reaction towards [Ta@Ge_8_As_4_]^3–^.

**Figure 5:**
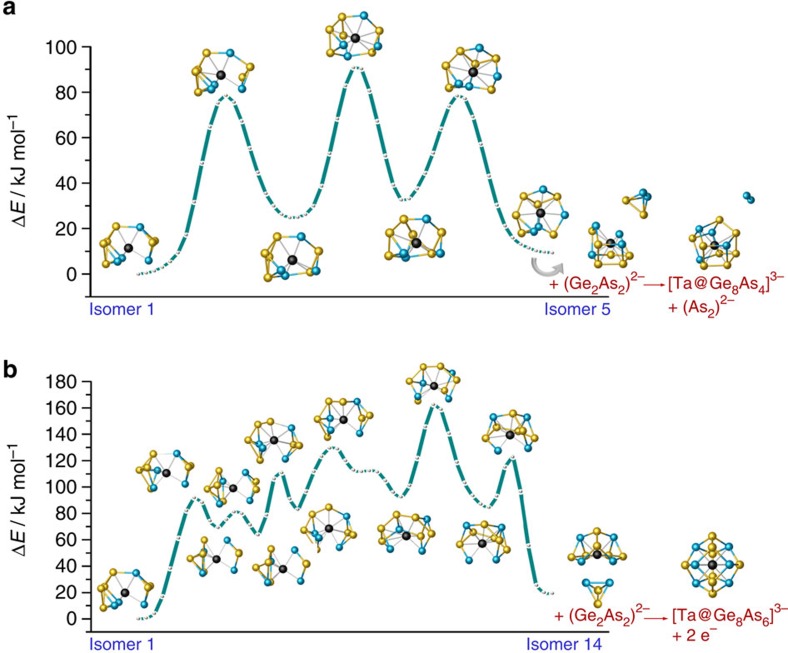
Stepwise re-arrangements of the most stable anion isomer in 3. (**a**) Re-arrangement into isomer 5. (**b**) Rearrangement into isomer 14. The final topologies are suitable for uptake of another (Ge_2_As_2_)^2−^ unit from **1** to form the clusters [Ta@Ge_8_As_4_]^3−^ (top) or [Ta@Ge_8_As_6_]^3−^ (bottom) in **4**. Detailed explanations are given in the text.

For the described steps, it remained to find possible pathways between the global minimum of [Ta@Ge_6_As_4_]^3–^, our ‘start structure', and the two most promising reactive species, the fifth and the fourteenth-stable isomer, called ‘end structures'. Here, we started from an initial pathway consisting of 14 intermediate structures obtained from interpolation between start and end structure, followed by an iterative optimization; in each iteration the gradients are calculated for each of the 14 structures. The number of possible pathways between any two isomers of [Ta@Ge_6_As_4_]^3–^ amounts to 6!·4!=17,280, as there are 6! ways to connect corresponding the 6 Ge atoms of the start and end structure, and similarly 4! ways for the 4 As atoms. For the two isomerizations, initial pathways were calculated for all possibilities; however, the costly optimization procedure was only carried out for those with interatomic distances not shorter than 1.6 A for all atoms in all interpolated structures of the initial pathways. The discarded pathways will most probably not be favourable in the end, and furthermore, quantum-chemical calculations for atom distances far away from equilibrium are often problematic. For the remaining ca. 700 pathways (for each of the two isomerizations), 35 iterations were carried out with economic DFT settings BP86 (refs 44, 45)/def-SVP (refs 46, 47, 48). Thereupon, the pathways with lowest barriers were refined with a larger number (53) of intermediate structures at the more accurate level TPSS/dhf-TZVP (refs 52, 53, 54) until convergence. For each of the two isomerizations, the finally resulting pathway with lowest barriers is shown in Fig. 5. Isomer 5 (fragment of [Ta@Ge_8_As_4_]^3–^) can be reached from the global minimum via three barriers with heights of 76/63/46 kJ mol^−1^. For isomer 14 (fragment of [Ta@Ge_8_As_4_]^3–^), the first barrier amounts to 92 kJ mol^−1^, followed by five barriers of 14/50/48/72/40 kJ mol^−1^. All barriers are obviously high enough to allow for (low yield) crystallization of the [Ta@Ge_6_As_4_]^3–^ anion in **3**, but also low enough to be finally overcome at the isomerization into suitable fragments as precursors of the two clusters in **4**. Interestingly, no direct pathway was found between isomer 5 and isomer 14, thus indicating that the subsequent reactions into the global minimum structures of [Ta@Ge_8_As_4_]^3–^ or [Ta@Ge_8_As_6_]^3–^, respectively (Supplementary Figs 29 and 30), are straight forward then.

One of the most important lessons we learned from these investigations is surprisingly obvious in its retrospective. Different from the preliminary assumption, and probably different from further suggestion on cluster formation pathways to be found in the literature, we deduce the following, general statement from our findings: clusters that are observed in crystalline ‘intermediates' are not necessarily directly involved in further cluster growth, but they can play a key role in the reaction cascade upon isomerization, such as found in the present case. Here, several intermediate compositions co-exist in nearly isoenergetic isomeric forms, with higher-energy isomers being naturally more reactive—thus not being detectable or at least not isolable, and most probably being the species that are actually involved in the reaction cascade, whereas the global minimum species rather represent thermodynamic sinks. We note in passing that the exact tracing of the re-arrangement between the respective isomers was facilitated by the presence of two different elements in the present case.

**Figure 6:**
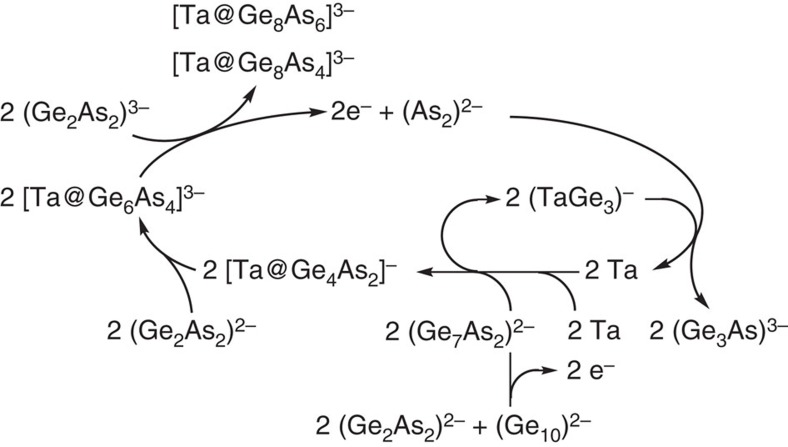
Formation cycle for the generation of Ta/Ge/As clusters. The scheme gives an overview of atom and electron balances along the pathway for the formation of multimetallic clusters [Ta@Ge_8_As_6_]^3−^ and [Ta@Ge_8_As_4_]^3−^ starting out from (Ge_2_As_2_)^2−^, (Ge_10_)^2−^ and Ta.

With respect to the known charges of the involved species, a cascade of redox steps needs to be taken into consideration. A final overview of atom and electron balances along the pathway is provided in Fig. 6. Following our assumptions, we can explain the whereabouts of all electrons during the complex process—except for two electrons to be released at the initial step, which does not seem to be problematic regarding the variety of small polyanionic by-products formed alongside the reaction, detectable by means of ESI-mass spectrometry (Supplementary Figs 14,18 and 24).

In summary, our findings strongly suggest that the transition metal atoms come into play early in the cluster formation process, that they are redox-active if delivered as neutral atoms, and that they are needed to weaken the bonds between the main-group atoms for easier fragmentation in their oxidized form. This clearly serves to explain the role of the transition metal atoms in such reactions in general, such that they can be viewed as catalysts triggering bond-breaking and bond-formation steps during the observed re-arrangements. Different from the behaviour of a common catalyst, at least a fraction of the transition metal atoms rather act as templates, such that they are finally surrounded by a rather stable, closed cluster shell, while another fraction seems to behave like a veritable catalyst, being involved in the formation of small fragments, which are anticipated to leave the formation pathway for being re-used again. Hence, we show that at least for the considered cluster family, the main-group atom shell is rather built stepwise around the transition metal atom instead of a transition metal atom entering a pre-formed, entire cluster shell.

## Discussion

With this study, we contributed to a more detailed and at least semi-quantitative understanding of ligand-free, multimetallic cluster formation by providing deeper insight into possible processes. This was possible by a combination of new experimental findings on binary and ternary anionic clusters with comprehensive quantum-chemical investigations applying a new methodology (GA-RP)^28^. We identified several isomers of a previously unknown key synthon, which are topologically identical with exact fragments of all known non-deltahedral intermetalloid clusters containing tetrel atoms. Additionally, the study rationalized why all of the reactions of (E^14^_2_E^15^_2_)^2–^ anions pass the (E^14^_7_E^15^_2_)^2–^ step, and why and how these, as well as reactions that start out from (E^14^_9_)^4–^, can proceed toward anions of the quoted type and composition by thorough screening of the energy hypersurface. This allowed shedding light on the formation of multimetallic clusters in general, not only for the compounds presented herein.

Our study still leaves room for further extension. In this regard, alternative pathways have to be explored that lead into other cluster families, such as the deltahedral ones. The uncountable variety of isomers with larger atom numbers and their re-arrangements into each other via energy barriers are likewise part of an exhaustive ongoing study, which has now become realistic by the development of the efficient methodology. The technique is applicable to all other systems dealing with binary or multinary cluster compounds, hence allowing for a detailed insight into cluster formation pathways. This way, we hope to finally contribute to a comprehensive understanding of inorganic reaction mechanisms in general, and to illuminate the former ‘black box'.

## Methods

### General synthetic methods

All manipulations and reactions were performed under dry Ar atmosphere by using standard Schlenk or glovebox techniques. All solvents were dried and freshly distilled prior to use. [2.2.2]crypt (4,7,13,16,21,24-Hexaoxa-1,10-diazabicyclo[8.8.8]hexacosane, purchased as Kryptofix 222 from Merck) was dried *in vacuo* for at least 18 h. The synthesis of ternary phases K_1_Ge_1_As_x_ (*x*=0.5, 1) were approached by fusing K, Ge and As in the respective stoichiometric ratios in a silica glass ampoule with an oxygen torch or in a tantalum ampoule within an oven at 950 °C, respectively.

### Fusion reactions

The generation of a solid mixture with the nominal composition of ‘KGeAs' was approached by combining K, Ge and As in equimolar amounts in a tantalum ampoule, which was sealed by arc-welding within the glove box. The ampoule was then placed in an oven for 48 h with initial heating to 950 °C and subsequent slow cooling to room temperature (heating and cooling rates of 50 K h^−1^). The resulting solid was thoroughly ground in a mortar prior to further use As confirmed by EDX spectroscopy results (Supplementary Fig. 1a and Supplementary Table 1), the solid product contains ∼4 atom-% of Ta. For this, the precursor phase will be denoted as ‘KGeAs:Ta' (precursor phase 1) in the following. The synthesis of the ‘KGeAs' solid was also performed in a silica glass ampoule with an oxygen torch (precursor phase 2). Results of EDX spectroscopy are provided in Supplementary Fig. 1b and Supplementary Table 1.

### Conjoint synthesis of 1–4

A total of 150 mg (0.81 mmol) of ‘KGeAs:Ta' (precursor phase 1) and 460 mg (1.22 mmol) of [2.2.2]crypt were weighed out into a Schlenk tube. Then 1,2-diaminoethane (*en*, 4 ml) was added to result in a dark red suspension. The reaction mixture was allowed to stir for 2 days. The liquid was filtered through a standard glass frit, yielding a red solution that was carefully layered by toluene (*tol*, 7 ml). Crystals of **1** form within 3 days. After 10 days, four distinct kinds of crystals (**1–4**) can be identified in the Schlenk tube (Supplementary Fig. 2). Due to the variety of crystals obtained in this reaction specific yields for **1–4** could not be determined with certainty. The overall yield of crystalline material is ∼19% (based on [2.2.2]crypt). [K([2.2.2]crypt)]_5_[K([2.2.2]crypt)(*en*)][Ta@Ge_8_As_4_]_1.21_[Ta@Ge_8_As_6_]_0.79_·*en* (**4**) is the major product of this reaction according to visual inspection of the Schlenk tube (∼60% of crystalline material).

### Conjoint synthesis of 1 and 2

For proving that the presence of Ta is not mandatory for the formation of salts of the (Ge_2_As_2_)^2–^ and (Ge_7_As_2_)^2–^ anion, 150 mg (0.81 mmol) of ‘KGeAs' prepared in a silica glass ampoule (precursor phase 2) and 460 mg (1.22 mmol) of [2.2.2]crypt were weighed out into a Schlenk tube and then suspended in *en* (4 ml). The reaction mixture was allowed to stir for 1 day. The orange mixture was filtered through a standard glass frit, and the resulting solution was carefully layered by *tol* (7 ml). After 7 days, black-looking, block-like crystals of **2** as well as orange plates of **1** were obtained. On cutting the dark blocks into a size suitable for single-crystal X-ray diffraction, they split off into orange plates (Supplementary Fig. 2, centre), which had only been agglomerated. Due to the crystal mixture, specific yields for **1** and **2** could not be determined with certainty. The overall yield of crystalline material is ∼24% (based on [2.2.2]crypt).

### Energy dispersive X-ray spectroscopy analyses

EDX analyses were performed to support the elemental composition that was suggested based on the single-crystal X-ray diffraction experiments. These were carried out using an EDX-device Voyager 4.0 of Noran Instruments coupled with an electron microscope CamScan CS 4DV. Data acquisition was performed with an acceleration voltage of 20 kV and 100 s accumulation time. The radiation emitted by the atoms was analysed: K-K, Ge-K, As-K and Ta-M/L. To minimize surface effects in the measurement, the K-lines were preferably used to calculate the elemental composition.

The spectra measured on the precursor phases 1 and 2 are shown in Supplementary Fig. 1, the results are summarized in Supplementary Table 1. Supplementary Table 2 summarizes the results for the precursor phases and compounds **1–4**, respectively. The results of the EDX investigations confirm the element ratios of the investigated substances within the expected accuracy.

### Crystallographic study of 1–4

Single-crystal X-ray diffraction data were collected on STOE imaging plate systems IPDS2 or IPDS2T, using graphite-monochromized Mo-Kα radiation (*λ*_Mo–Kα_=0.71073 Å) at 100 K. The structures were solved by direct methods, using SHELXS-97 (ref. 60) or SIR2011 (ref. 61), and refined by full-matrix-least-squares methods against *F*^2^ with SHELXL-2013 software^60^. Crystal data: (**1**) C_38_H_80_As_2_Ge_2_K_2_N_6_O_12_, *M*_r_=1186.3, triclinic, space group *P*1, *a*=10.9738(4) Å, *b*=11.9313(4) Å, *c*=12.6146(6) Å, *α*=118.021(3)°, *β*=108.361(4)°, *γ*=96.476(3)°, *V*=1,315.07(10) Å^3^, *Z*=1, *ρ*_calc_=1.498 g cm^−3^, *μ*(Mo_K*α*_)=2.608 mm^−1^, 23,683 reflections were measured, 10,275 of which were unique, *R*(int)=0.065, *R*_1_ (*I*>2*σ*(*I*))=0.0454, *wR*_2_ (all data)=0.1189, *S* (all data)=1.075. (**2**) C_36_H_72_As_2_Ge_7_K_2_N_4_O_12_, *M*_r_=1,489.14, trigonal, space group 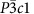, *a*=11.8653(3) Å, *c*=22.3848(9) Å, *V*=2729.2(2) Å^3^, *Z*=2, *ρ*_calc_=1.812 g cm^–3^, *μ*(Mo_K*α*_)=5.212 mm^–1^, 3,581 reflections were measured, 1,942 of which were unique, *R*(int)=0.035, *R*_1_ (*I*>2*σ*(*I*))=0.0521, *wR*_2_ (all data)=0.1048, *S* (all data)=0.958. (**3**) C_68_H_124_As_4_Ge_6_K_3_N_6_O_18_Ta, *M*_r_=2347.19, monoclinic, space group *P*2_1_/*n*, *a*=13.7543(2) Å, *b*=28.6077(5) Å, *c*=22.9845(4) Å, *β*=92.489(1)°, *V*=9,035.4(3) Å^3^, *Z*=4, *ρ*_calc_=1.725 g cm^–3^, *μ*(Mo_K*α*_)=4.829 mm^−1^, 148,425 reflections were measured, 19,185 of which were unique, *R*(int)=0.070, *R*_1_ (*I*>2*σ*(*I*))=0.0346, *wR*_2_ (all data)=0.0838, *S* (all data)=0.884. (**4**) C_56_H_116_As_4.79_Ge_8_K_3_N_8_O_18_Ta, *M*_r_=2,427.40, triclinic, space group 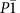, *a*=16.6082(5) Å, *b*=22.6960(8) Å, *c*=23.9068(8) Å, *α*=94.960(3)°, *β*=94.036(3)°, *γ*=91.402(3)°, *V*=8951.2(5) Å^3^, *Z*=4, *ρ*_calc_=1.801 g cm^–3^, *μ*(Mo_K*α*_)=5.819 mm^–1^, 64,236 reflections were measured, 31,253 of which were unique, *R*(int)=0.057, *R*_1_ (*I* > 2*σ*(*I*))=0.0735, *wR*_2_ (all data)=0.1973, *S* (all data)=0.990. Further details are given in Supplementary Table 3. Cambridge Crystallographic Data Centre deposition codes are provided in the Accession codes section.

### Details of the structure determination of 1

The structure of [K([2.2.2]crypt)]_2_[Ge_2_As_2_]·*en* (**1**) comprises two independent [K[2.2.2]crypt)]^+^ cations, a tetrahedral (Ge_2_As_2_)^2–^ anion (Supplementary Fig. 3) and a disordered *en* molecule. The space group is indeed *P*1 with pseudo-symmetry 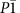. The tetrahedral (Ge_2_As_2_)^2–^ anion breaks the centro-symmetry. At *Z*=1 it would appear disordered on the atomic positions of a cube in case of true centro-symmetry, which is not the case here. Since Ge and As cannot be distinguished by X-ray diffraction using Mo-Kα radiation, all four sites are half-occupied by Ge and As. Supplementary Table 4 summarizes interatomic distances and angles. The cations form a honeycombe-like packing with channels along *a* in which the (Ge_2_As_2_)^2–^ anions are aligned together with the disordered *en* molecules (Supplementary Fig. 4).

### Details of the structure determination of 2

Compound [K([2.2.2]crypt)]_2_[Ge_7_As_2_] (**2**) crystallizes in space group 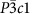 with the [K[2.2.2]crypt)]^+^ cation on a threefold axis (site 3d) and the (Ge_7_As_2_)^2−^ anion on the 2a site with symmetry 32 (*D*_3_). The Ge/As occupation 7:2 as confirmed by mass spectrometry (MS) and density functional theory (DFT) investigations was fixed for each cluster atom. Around the 2a site, 24 metal positions could be located. This can be explained by orientational disorder of a 9-atom anion cluster (Supplementary Fig. 5a) over three positions generated by the threefold axis along [001] with Ge/As1 common for two orientations (Supplementary Fig. 5b). The large displacement ellipsoids let us assume that the *C*_2_ axis (or *C*_4_ axis with indistinguishable Ge/As) of the (Ge_7_As_2_)^2–^ anion does not coincide exactly with the *C*_2_ axes of the crystallographic 2a site. Thus the disorder model is really an overlay of six instead of three orientations and the refined geometry may appear adulterated, therefore. Supplementary Table 5 summarizes interatomic distances and angles. Similar to compound **1**, but with trigonal symmetry, the cations form a honeycombe-like packing with the disordered anions in channels along the *c* axis (Supplementary Fig. 6).

### Details of the structure determination of 3

The structure of [K([2.2.2]crypt)]_3_[Ta@Ge_6_As_4_]·*2tol* (**3**) in space group *P*2_1_/*n* is built by a [Ta@Ge_6_As_4_]^3–^ anion showing statistical overlay of two isomers with occupations of 87.7(1) and 12.3(1)% (Supplementary Fig. 7, Supplementary Table 6), three independent [K([2.2.2]crypt)] cations (Supplementary Fig. 8) and two toluene solvent molecules. The interpretation of the disorder model of the anion isomers and attribution of Ge and As to the atom sites has been done based on theoretical calculations. The large anisotropic displacement parameters of Ge7A, Ge8A and Ge9A suggest additional orientational disorder of the tetrahedral (Ge_3_As)^3–^ group of isomer 2. The bond lengths in this region may therefore be adulterated. The left hand side of the isomer might derive from an intermediately formed (Ge_6_As_3_)^–^ nine-atom cage, homologues of which had been observed in previous studies with Sn/Bi or Pb/Bi anions^25^,^26^,^34^. The formation of (Ge_6_As_3_)^–^, in turn, is attributed on another relative orientation of (Ge_2_As_2_)^2–^ and (Ge_10_)^2–^ during the respective attack in the first step of the reaction cascade. The complicated packing of cations, anions and toluene molecules in compound **3** is shown in Supplementary Fig. 9.

### Details of the structure determination of 4

The triclinic centrosymmetric structure of [K([2.2.2]crypt)]_5_[K([2.2.2]crypt)(*en*)][Ta@Ge_8_As_4_]_1.21_[Ta@Ge_8_As_6_]_0.79_·*en* (**4**) shows severe disorder effects on the two independent anion positions as well as at some of the six independent cations. The anions are on two independent sites. On site one, superposition of a [Ta@Ge_8_As_4_]^3−^ cluster (89.4%) and a [Ta@Ge_8_As_6_]^3−^ cluster (10.6%) has been found. The occupations by Ge and/or As were taken according to information from EDX spectroscopy, mass spectrometry, the most stable configurations of DFT calculations and their probable disordered orientations (Supplementary Fig. 10). Bond lengths are given in Supplementary Table 7. On site 2, a [Ta@Ge_8_As_6_]^3−^ cluster is dominating (68.5%) superimposed by a [Ta@Ge_8_As_4_]^3−^ cluster in two different orientations (12.9 and 18.6%). By this complicated disorder with many approximately common positions, the individual geometrical data appear adulterated and are not listed, therefore. The structures are given in Supplementary Fig. 11. Five of the six cations are [K[2.2.2](crypt)] cations like in the structures of compounds **1–3**. The refinement of these [2.2.2]crypt molecules in the presence of many heavy atoms was performed using geometrical restraints on the bond lengths and 1,3- distances. The anisotropic displacement ellipsoids show sometimes irregular shape as they include disorder effects (Supplementary Fig. 12a–d). They were refined with restraints to avoid too anisotropic displacement parameters. For one [2.2.2]crypt ligand with strong disorder, no sensible disorder model could be established. Its contribution was subtracted, therefore, by the back Fourier transform method from the data set. A sixth cation has an *en* molecule coordinated to K^+^ in addition to the [2.2.2]crypt ligand (Supplementary Fig. 12e). In addition, a non-coordinated *en* molecule was located. The packing of molecules in the structure of compound **4** is shown in Supplementary Fig. 13.

### Electrospray ionization mass spectrometry investigations

ESI-MS measurements have been performed on a Finnigan LTQ-FT spectrometer by Thermo Fischer Scientific in the negative ion mode: Spray voltage 3.90 kV, capillary temperature 300 °C, capillary voltage –11 V, tube lens voltage –140 V, sheath gas flow rate 25 arb, sweep gas flow rate 0 arb. As it is common for Zintl anions and intermetallic cluster anions, the observed fragments have been detected as oxidized, singly charged species.

### ESI-MS of the *DMF/en/*[2.2.2]crypt extract of ‘KGeAs'

The ESI(–) mass spectrum of the extract of `KGeAs' (precursor phase 2, prepared in a silica glass ampoule) in DMF/*en* in presence of [2.2.2]crypt was measured after 18 h of extraction time (Supplementary Fig. 14). The study confirms the concurrent presence of the singly charged cluster species (Ge_2_As_2_H)—(m/z=296.69, Supplementary Fig. 15), obviously formed under ESI-MS conditions by protonation of the anion in **1,** as well as (Ge_7_As_2_)^–^ (m/z=658.29, Supplementary Fig. 16) side by side with (Ge_10_)^–^ (m/z=726.22, Supplementary Fig. 17) in the same solution. The concurrent presence of these polyanions strongly supports the assumption that the 9-atom cluster (Ge_7_As_2_)^2–^ is the product of the reaction of two (Ge_2_As_2_)^2–^ clusters with one (Ge_10_)^2–^. Also adducts of K^+^ and [K([2.2.2]crypt)]^+^ were detected: (Ge_7_As_2_K)^–^ (m/z=697.26), (Ge_10_C_18_H_36_N_2_O_6_K; m/z=1141.44).

### ESI-MS of the *en*/[2.2.2]crypt extract of ‘KGeAs:Ta'

The ESI(–) mass spectra of the extract of `KGeAs:Ta' (precursor phase 1, prepared in a Ta ampoule) in *en* in the presence of [2.2.2]crypt was measured after 3 h of extraction time. The overview spectrum (Supplementary Fig. 18) confirms the presence of a variety of clusters in solution. Remarkable is the presence anions of **1** and intermediate **3** in the solution after just 3 h of extraction. Due to fragmentation of the high-mass isotopic patterns and increasing degradation of the solution during injection the observed intensity of the isotopic pattern decreased during the measurement. In the reaction mixture, the presence of (Ge_2_As_2_H)^–^ (m/z=296.69) beside (Ge_3_As)^–^ (m/z=292.68) was confirmed (Supplementary Fig. 19). The isotopic patterns of (Ge_6_As_4_Ta)^–^ (m/z=916.17), (Ge_6_As_4_TaK)^–^ (m/z=955.13) and (Ge_6_As_4_TaK_2_)^–^ (m/z=994.09, Supplementary Figs 20–22) were identified as well as these of [K([2.2.2]crypt)]^+^ adducts. In addition, the isotopic pattern of (Ge_5_As_3_C_18_H_36_N_2_O_6_K_3_)^–^ (m/z=1082.5) was identified (Supplementary Fig. 23).

### ESI-MS of a DMF/*en* solution of 4

In the spectrum, various anions with and without [K([2.2.2]crypt)]^+^ were identified (Supplementary Fig. 24). The following species, representing oxidized clusters of [Ta@Ge_8_As_4_]^3–^ and [Ta@Ge_8_As_6_]^3–^ were found: (Ge_8_As_4_TaC_36_H_72_N_4_O_12_K_2_)^–^ (m/z=1892.46, Supplementary Fig. 25) and (Ge_8_As_6_TaC_18_H_36_N_2_O_6_K)^–^ (m/z=1627.07, Supplementary Fig. 26).

### Quantum-chemical methods

All calculations were done with the programme system TURBOMOLE (ref. 38). Global minimum searches were carried out with a DFT-based GA (refs 42, 43) extended^28^ by an atom-to-place re-assignment step^50^,^51^ with the following settings: Population size *P*=20 structures; cross-over after optimization of 10 structures, leading to the formation of *P*=10 new (child) structures. The mutation probability was set to 1%. The procedure was stopped after 30 generations. Optimizations of reaction pathways were done with an iterative method based on a local quadratic approximation of the energy hypersurface^29^. For these steps bases of polarized split-valence quality, def-SV(P)^46^,^47^ (with an effective core potential of Wood-Boring type for Ta (ref. 48) and the generalized gradient approximation DFT functional (BP86) by Becke^44^ and Perdew^45^ were chosen for reasons of economy. The resulting best structures and best pathways were re-optimized using more flexible polarized triple zeta valence basis sets dhf-TZVP (ref. 53; with an effective core potential of the Dirac-Hartree-Fock type for Ta)^54^ and the meta-generalized gradient approximation functional by Tao, Perdew, Staroverov and Scuseria (TPSS)^52^. For comparison, also PBE^56^ and TPSSh^57^ functionals were applied, with the results given in Supplementary Tables 8, 9 and 12. For all cases, the negative charges of the clusters were compensated by using the COSMO^49^, with the dielectric constant *ɛ* set to infinity (default). Thermochemical data were calculated from partition sums within the standard harmonic oscillator approximation for molecules in the gas phase^58^. The vibrational frequencies were used non-scaled. The RI approximation was used throughout^62^. Molecules were visualized with the programme CYLView^63^.

## Additional information

**Accession codes:** The X-ray crystallographic coordinates for structures reported in this Article have been deposited at the Cambridge Crystallographic Data Centre, under deposition numbers 1016136-1016139. These data can be obtained free of charge from The Cambridge Crystallographic Data Centre via www.ccdc.cam.ac.uk/data_request/cif.

**How to cite this article:** Mitzinger, S. *et al*. Understanding of multimetallic cluster growth. *Nat. Commun.* 7:10480 doi: 10.1038/ncomms10480 (2016).

## Supplementary Material

Supplementary Information Supplementary Figures 1-30, Supplementary Tables 1-12 and Supplementary References.

Supplementary Data 1 CIF of compound 1

Supplementary Data 2 CIF of compound 2

Supplementary Data 3 CIF of compound 3

Supplementary Data 4 CIF of compound 4
