# Supplementary material for: Understanding of multimetallic cluster growth
Source: Nat Commun. 2016 Jan 25;7:10480. doi: 10.1038/ncomms10480 (PMC4737759; doi:10.1038/ncomms10480)
Supplement: Supplementary Information — Supplementary Figures 1-30, Supplementary Tables 1-12 and Supplementary References. [file ncomms10480-s1.pdf]

Supplementary Figures

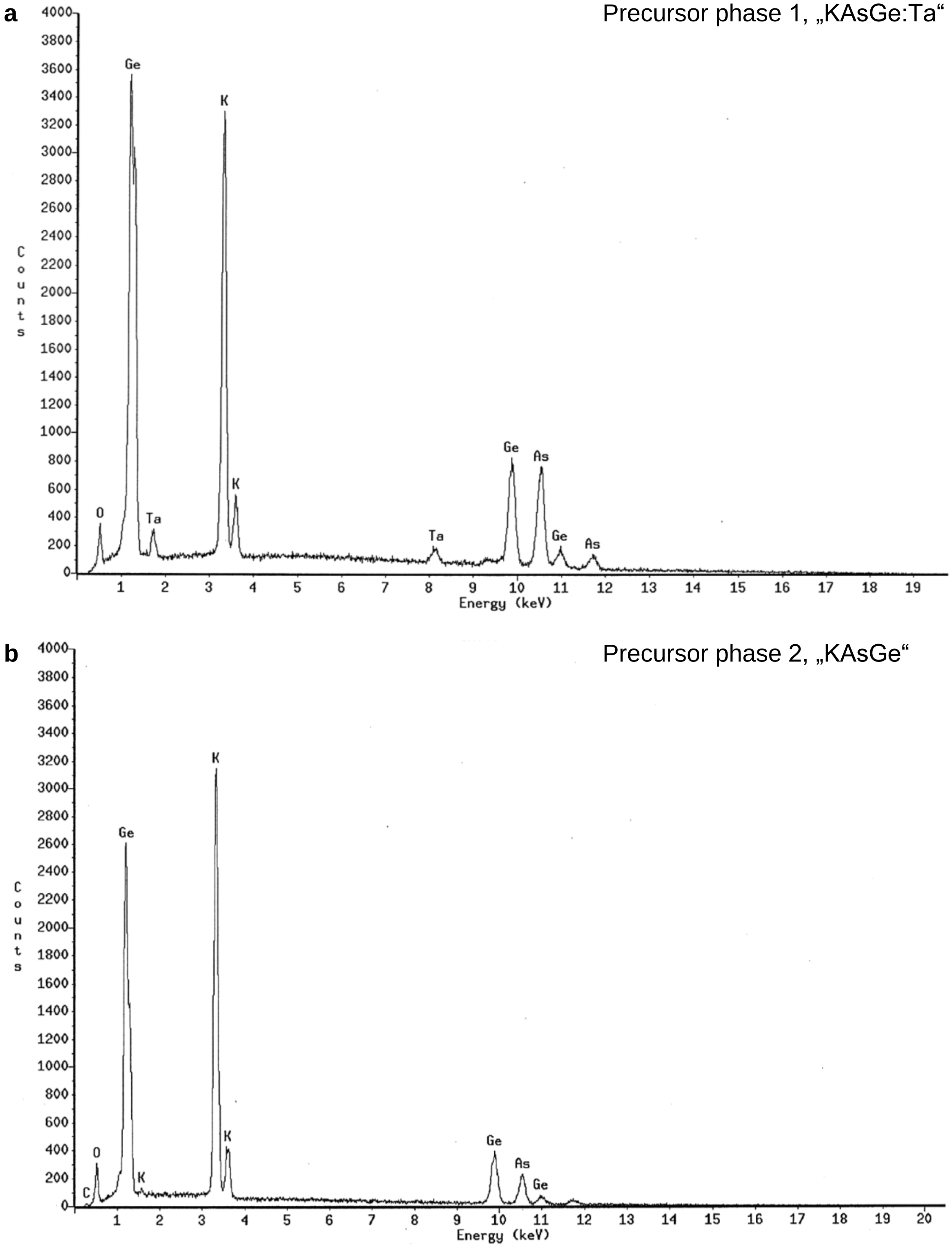

Supplementary Figure 1. Energy dispersive X-ray (EDX) spectra of the solid products. (**a**) Spectrum of the solid upon melting a K:Ge:As mixture (1:1:1) in a Ta ampoule (precursor phase 1. (**b**) Spectrum of the solid upon melting a K:Ge:As mixture (1:1:1) in a silica glass ampoule (precursor phase 2).

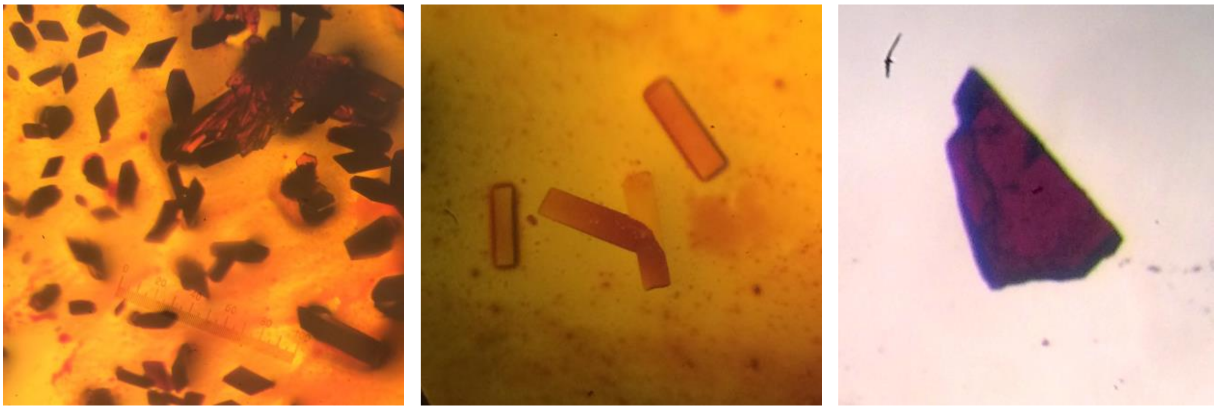

a **b** **c**

Supplementary Figure 2. (**a**) Rhombic, dark crystals of **4** along with stacked plate-shaped, red crystals of **1**. (**b**) Rectangular, orange-red crystals of **2** (center). (**c**) Red, block-type single crystal of **3** (right).

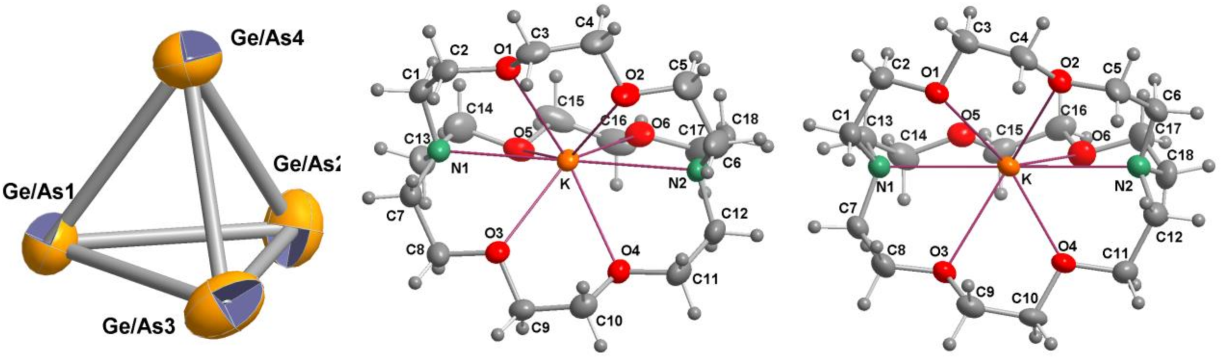

a **b** **c**

Supplementary Figure 3. (**a**) $(Ge_2As_2)^{2-}$ Anion in **1**. Interatomic distances and angles are given in Supplementary Table 4. (**b**) Cation 1 in **1**. (**c**) Cation 2 in **1**. Displacement ellipsoids are shown at the 50% probability level.

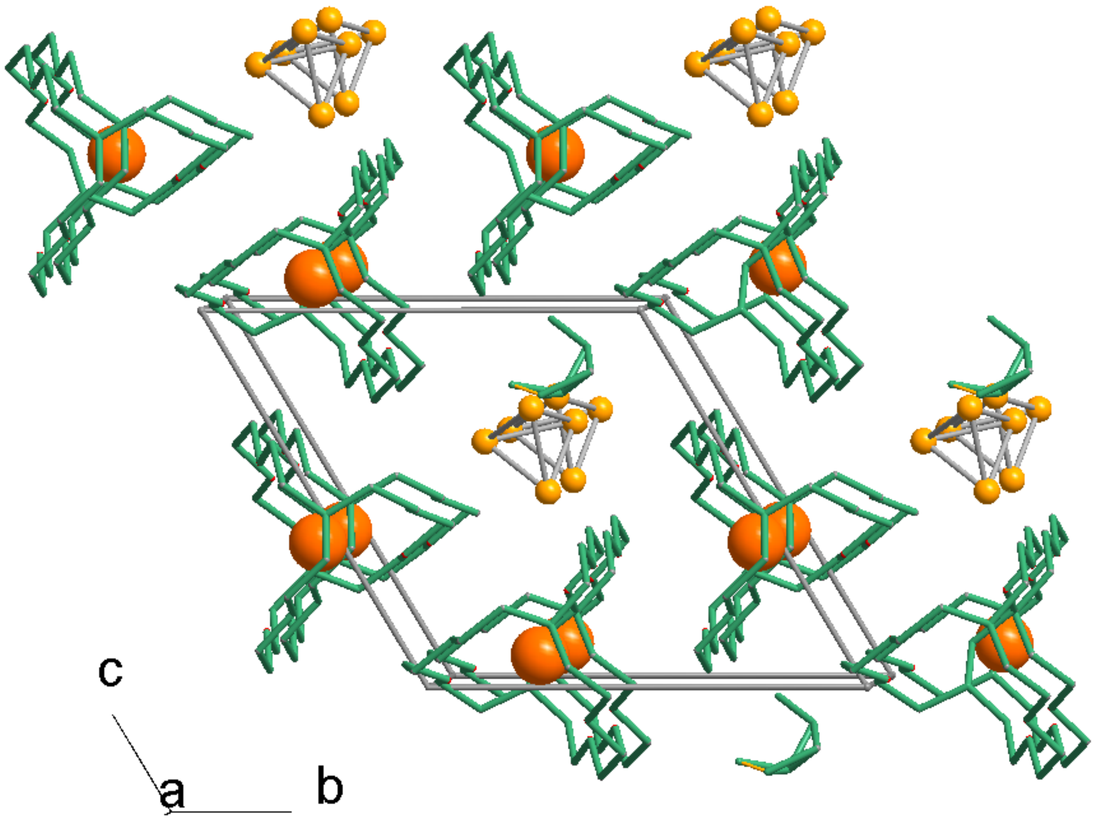

Supplementary Figure 4. Packing in the structure of **1**. Arbitrary radii: Ge/As yellow, K orange. [2.2.2]crypt ligands and *en* molecules are given as green wires.

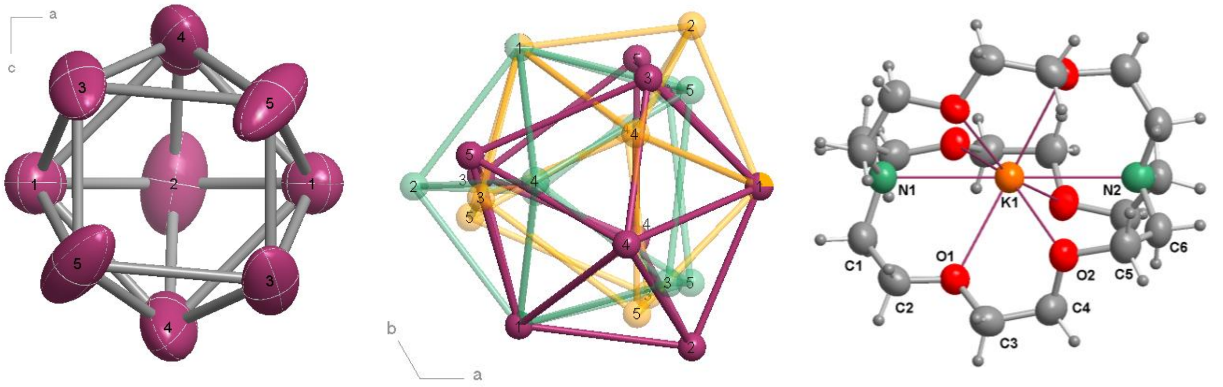

a **b** **c**

Supplementary Figure 5. (**a**) $(Ge_7As_2)^{2-}$ anion in **2** projected along the 2-fold axis. Displacement ellipsoids at the 50% probability level. Interatomic distances are given in Supplementary Table 5. (**b**) The three orientations of the anion generated by the 3-fold axis (arbitrary radii). (**c**) C_3-symmetric cation in **2**. Displacement ellipsoids are shown at the 50% probability level.

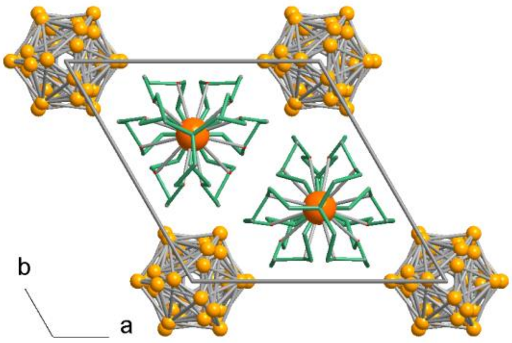

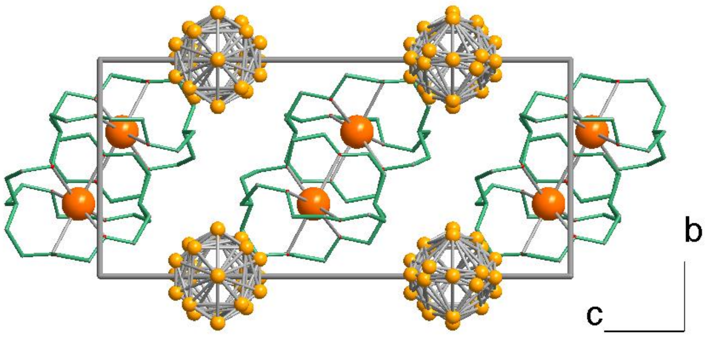

a **b**

Supplementary Figure 6. Packing of cations and anions in the structure of **2**. (**a**) Projection along [001]. (**b**) Projection along [100]. Ge, As and K atoms are shown with arbitrary radii, Ge/As yellow, K orange. [2.2.2]crypt ligands are shown as green wires.

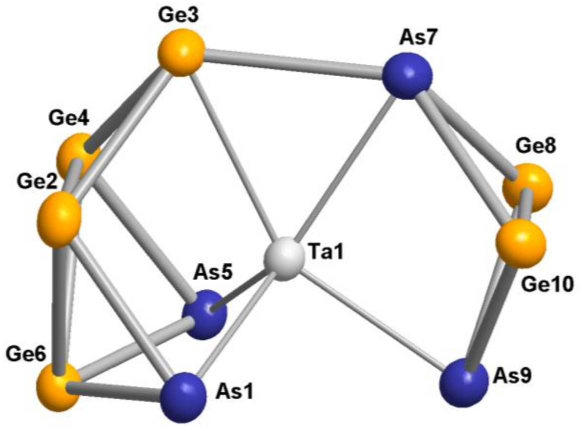

a

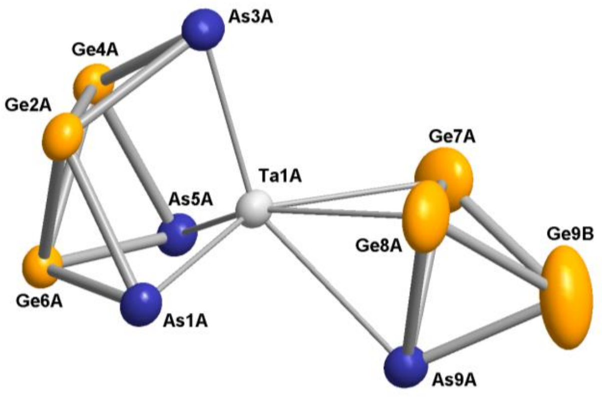

b

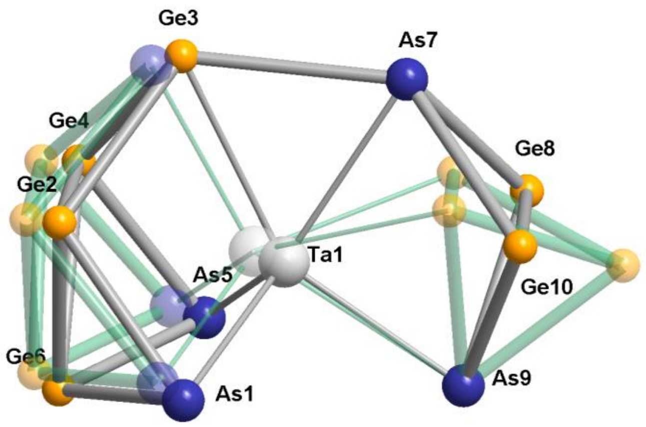

c

Supplementary Figure 7. (**a**) Isomer 1 (87.7%) of the $[TaGe_6As_4]^{3-}$ anion in **3**. (**b**) Isomer 2 (12.3%). Displacement ellipsoids at the 50% level. (**c**) Disorder model illustrated by superposition of both isomers, shown with arbitrary radii and with the second isomer in semi-transparent mode with green bonds. Interatomic distances are given in Supplementary Table 6.

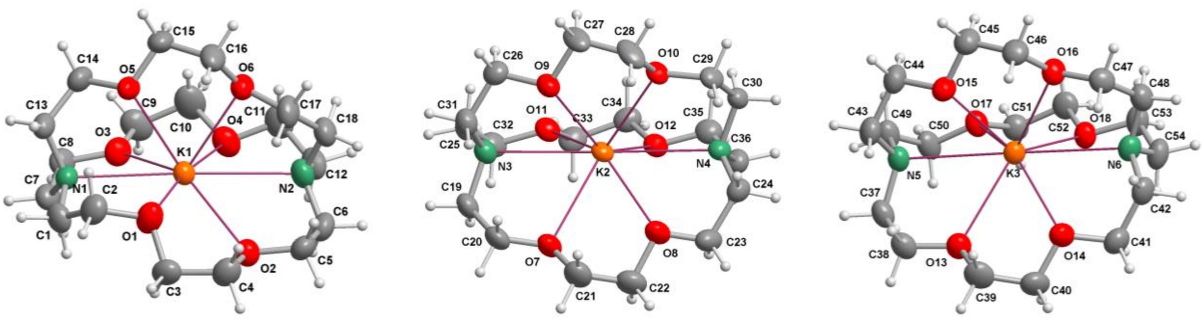

a **b** **c**

Supplementary Figure 8. The three independent [K([2.2.2]crypt)]$^+$ cations 1 (**a**), 2 (**b**), and 3 (**c**) in **3**. Displacement ellipsoids are shown at the 50% level.

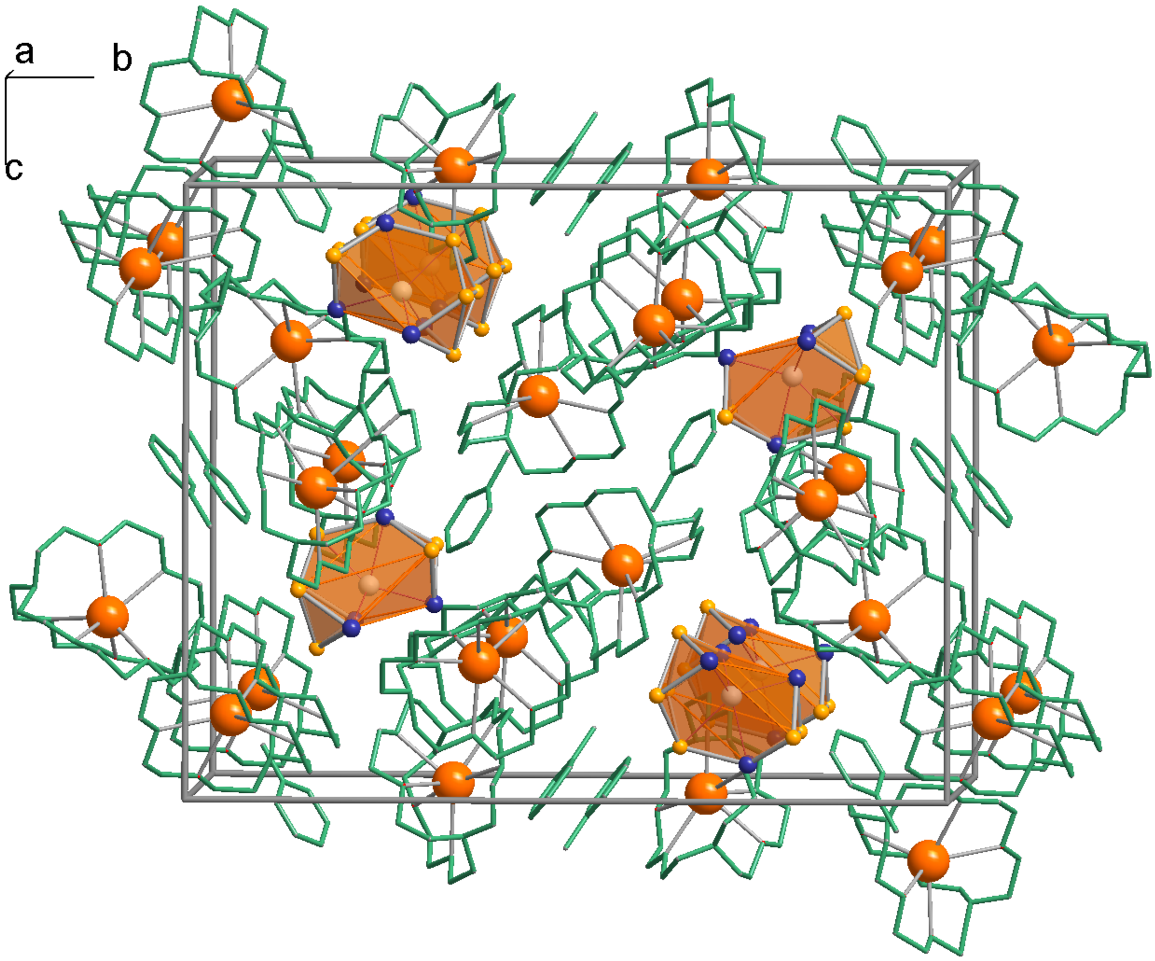

Supplementary Figure 9. Packing in the structure of compound **3**. Arbitrary radii: Ta grey, Ge yellow, As blue, K orange. [2.2.2]crypt ligands and toluene molecules are drawn as green wires.

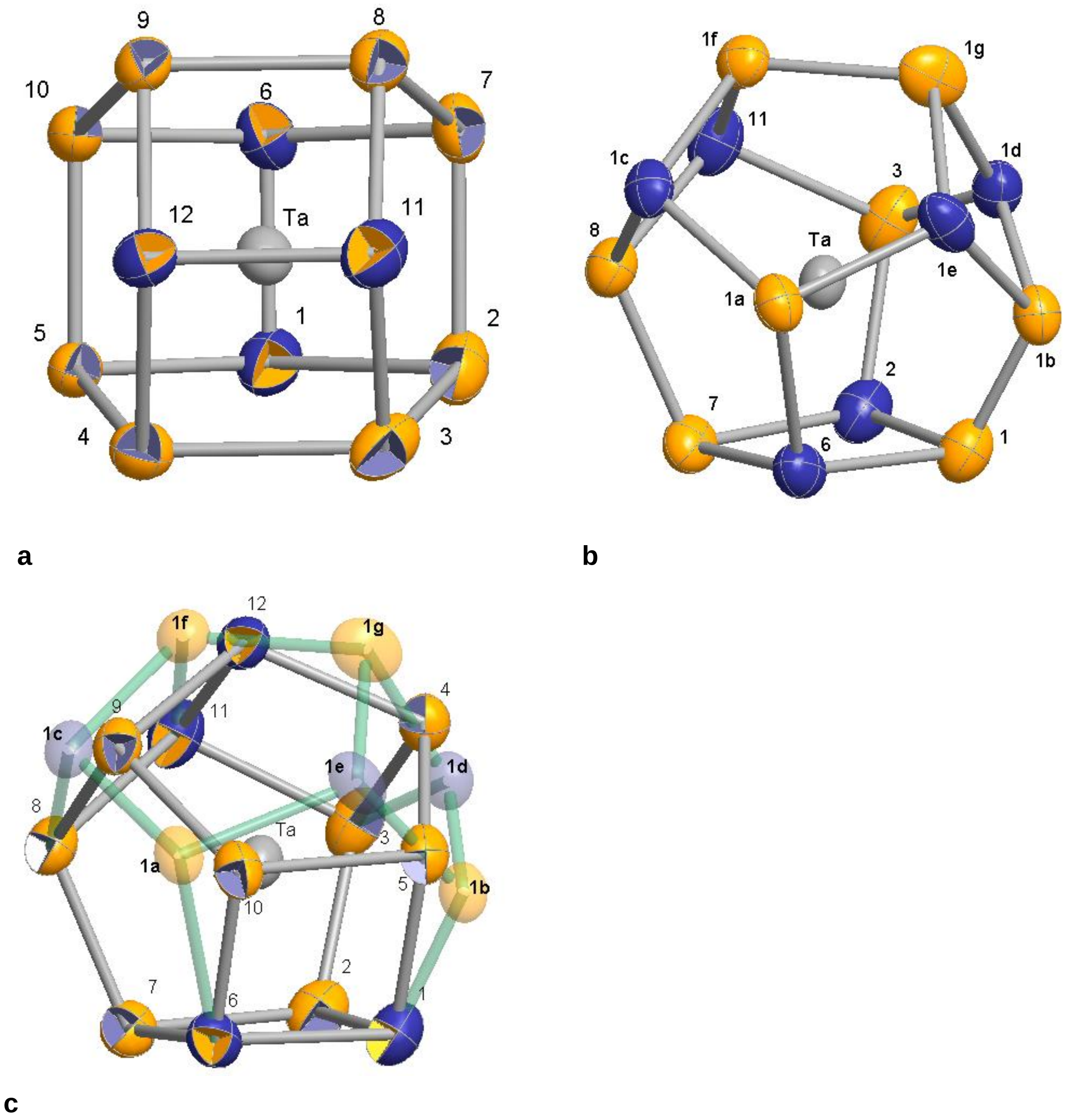

Supplementary Figure 10. Anion site 1 in **4**. (**a**) Cluster 1a $[Ta@Ge_8As_4]^{3-}$ with a 12-atom shell. Yellow surface: 75%Ge, 25%As; blue surface: 50%Ge, 50%As. (**b**) Cluster 1b $[Ta@Ge_8As_6]^{3-}$ with a 14-atom shell. Yellow: Ge, blue: As. (**c**) Disorder model illustrated by superposition of the two clusters, with the 12-atom shell drawn with grey bonds, and the 14-atom shell drawn in semi-transparent mode with green bonds. All displacement ellipsoids are shown at the 50% probability level. Interatomic distances are given in Supplementary Table 7.

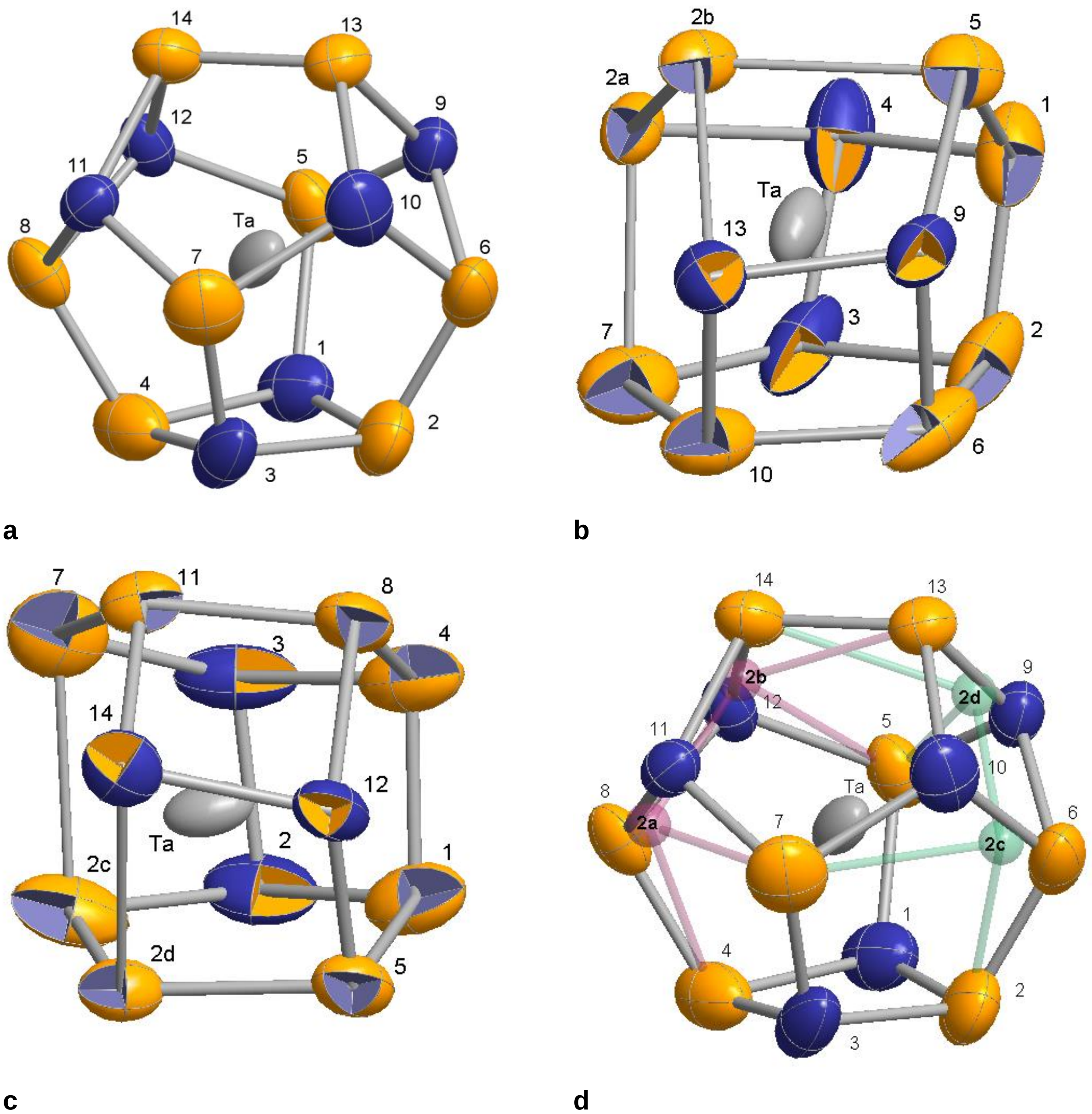

Supplementary Figure 11. Anion site 2 in **4**. (**a**) Cluster 2a $[Ta@Ge_8As_6]^{3-}$ with a 14-atom shell. (**b**) Cluster 2b $[Ta@Ge_8As_4]^{3-}$ with a 12-atom shell, first orientation. (**c**) Cluster 2c $[Ta@Ge_8As_4]^{3-}$ with a 12-atom shell, second orientation. (**d**) Disorder model illustrated by superposition of all three components. Red transparent: additional atoms for component 2b, Green transparent: additional atoms for component 2c. All displacement ellipsoids are shown at the 50% probability level. Color codes are the same as in Supplementary Figure 10.

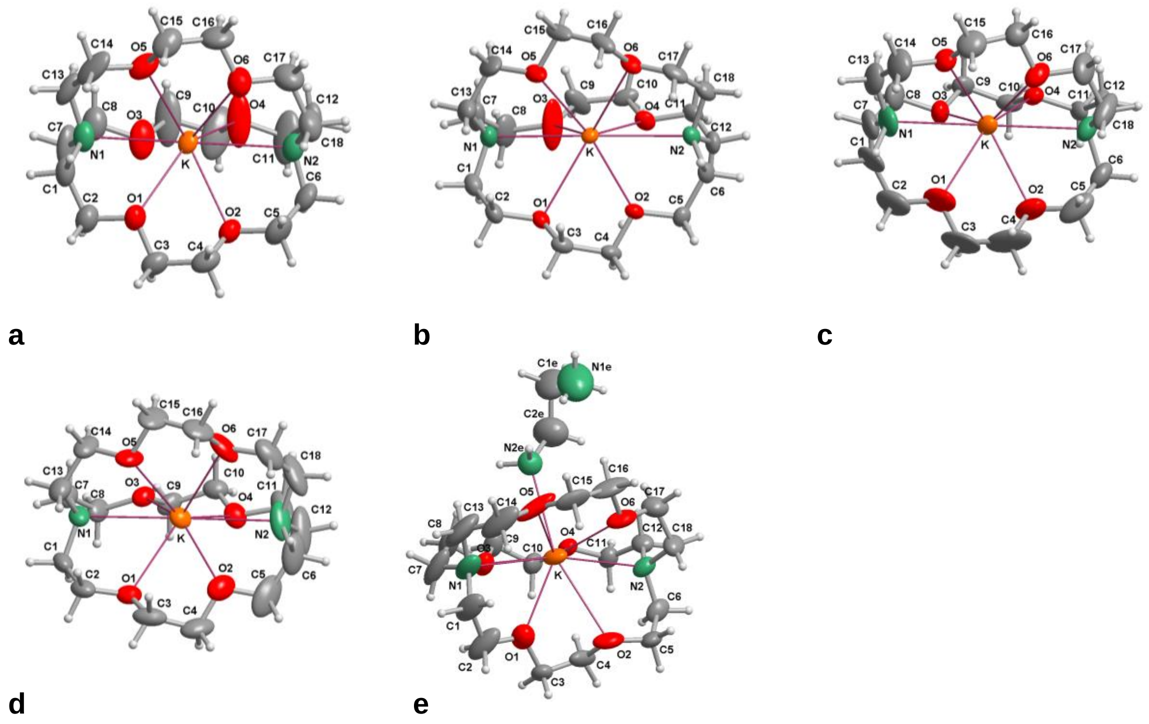

Supplementary Figure 12. The four localized [K([2.2.2]crypt)]$^+$ cations 1 (**a**), 2 (**b**), 3 (**c**), and 4 (**d**) of five cations in **4**. (**e**) The [K([2.2.2]crypt)(en)]$^+$ cation in **4**. H atoms at N1e are modelled with 2/3 occupation. All displacement ellipsoids are shown at the 30% probability level.

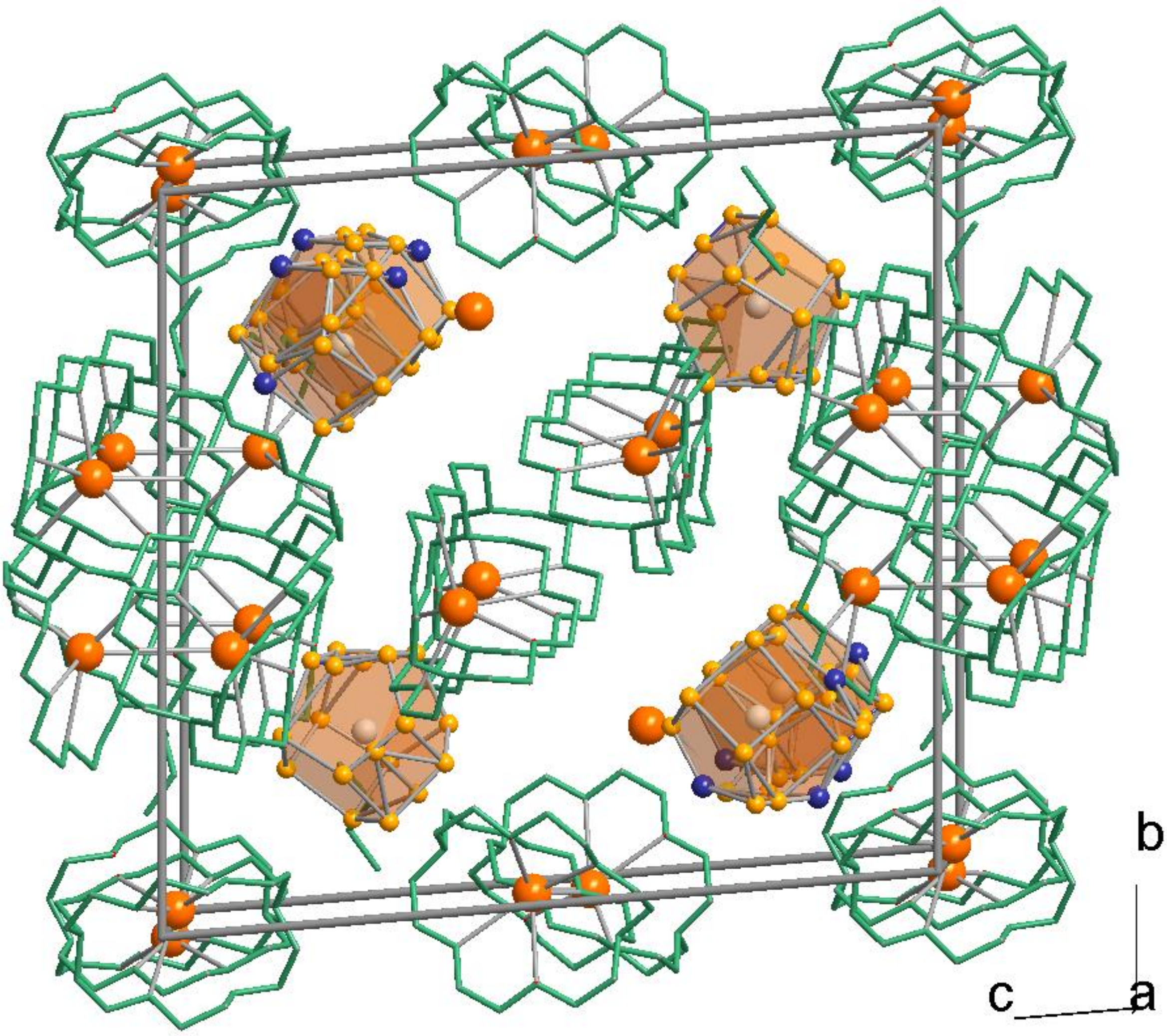

Supplementary Figure 13. Packing of cations and anions in the structure of compound **4** with arbitrary radii. Anionic clusters are shown as polyhedral. Ta grey, Ge yellow, As blue, K orange. [2.2.2]crypt ligands and *en* molecules are drawn as green wires. Note that one [2.2.2]crypt ligand was not modelled due to heavy disorder.

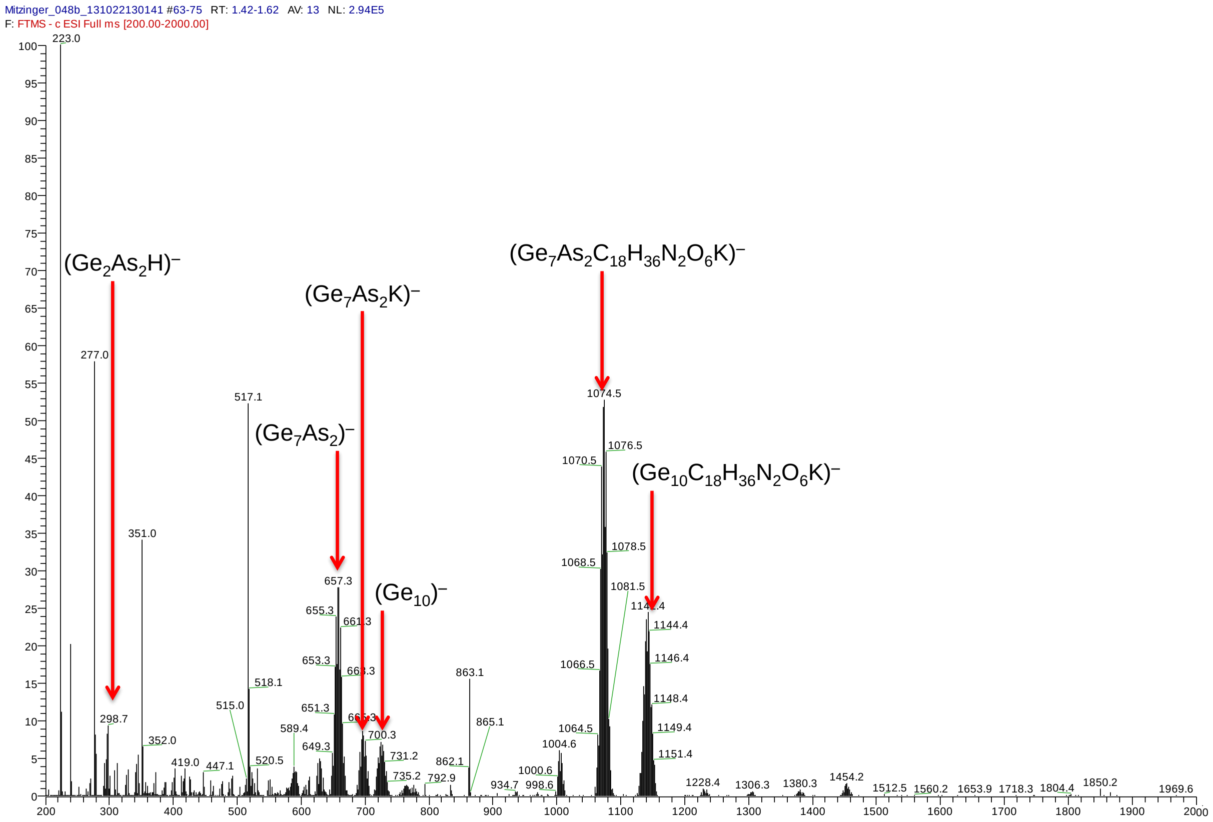

Supplementary Figure 14. ESI-MS(–) overview spectrum of the extract of "KGeAs" (precursor phase 2, prepared in a silica glass ampoule) in DMF/*en* in the presence of [2.2.2]crypt, shown between 200 and 2000 m/z.

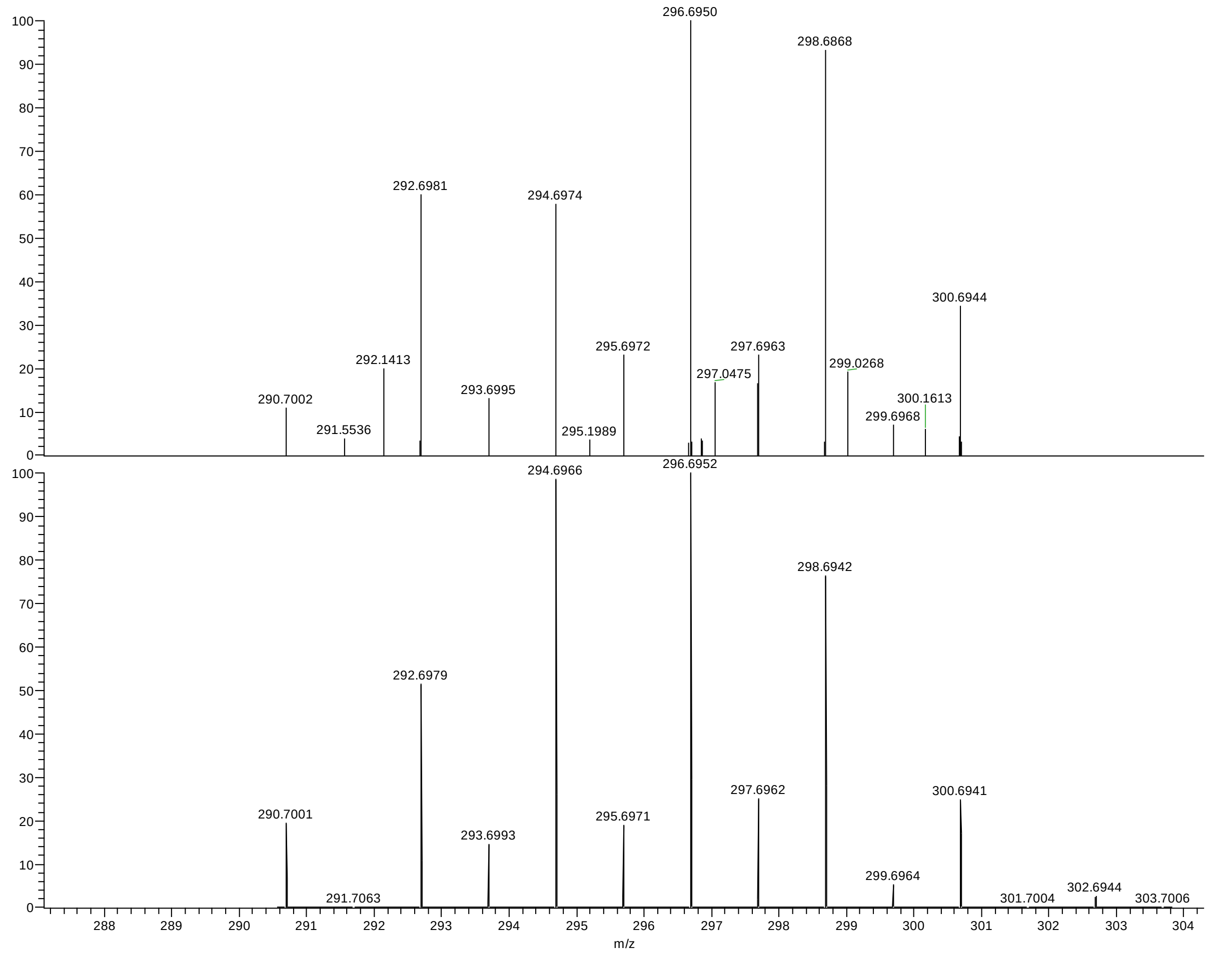

Supplementary Figure 15. ESI(–) mass peak of $(Ge_2As_2H)^-$. Measured (top) vs. calculated (bottom) spectrum.

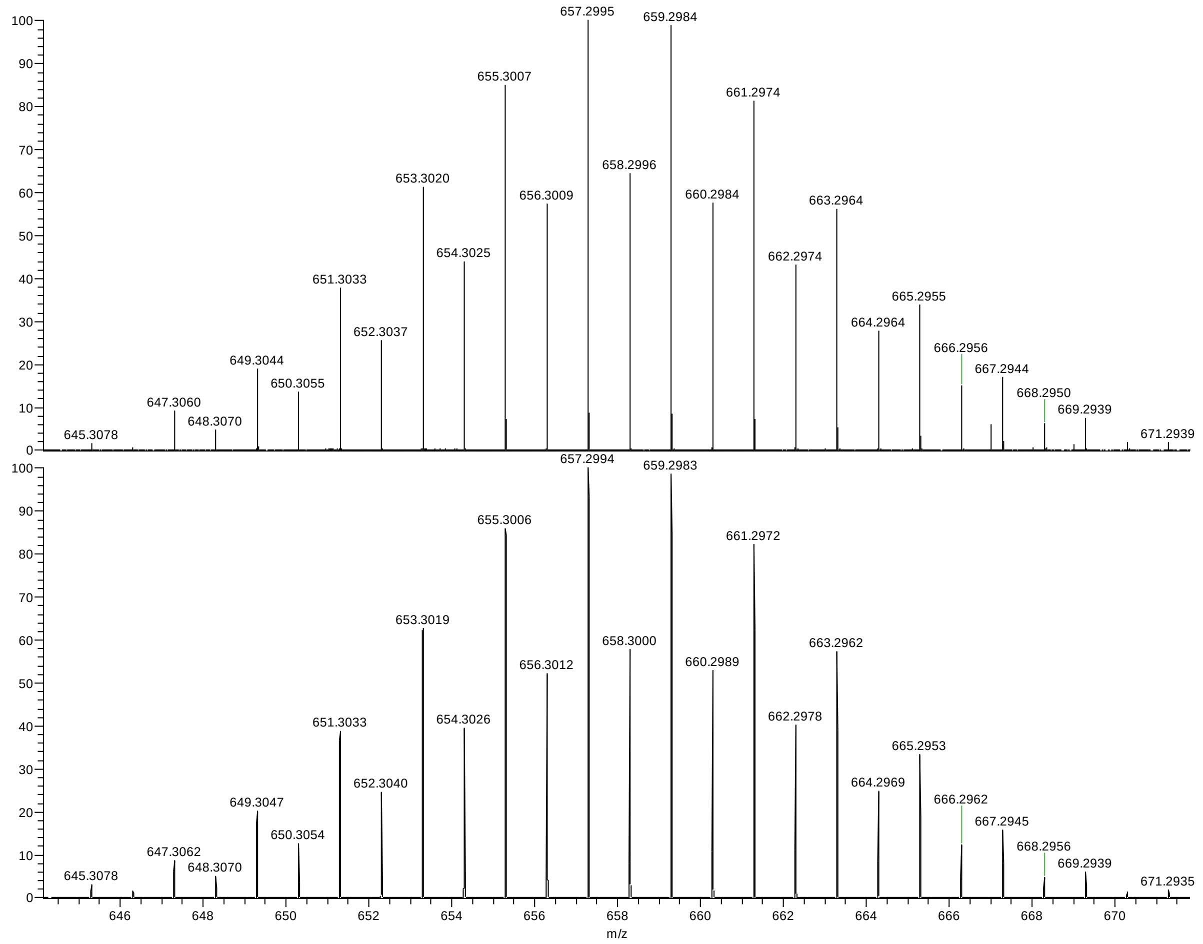

Supplementary Figure 16. ESI(–) mass peak of $(Ge_7As_2)^-$. Measured (top) vs. calculated (bottom) spectrum.

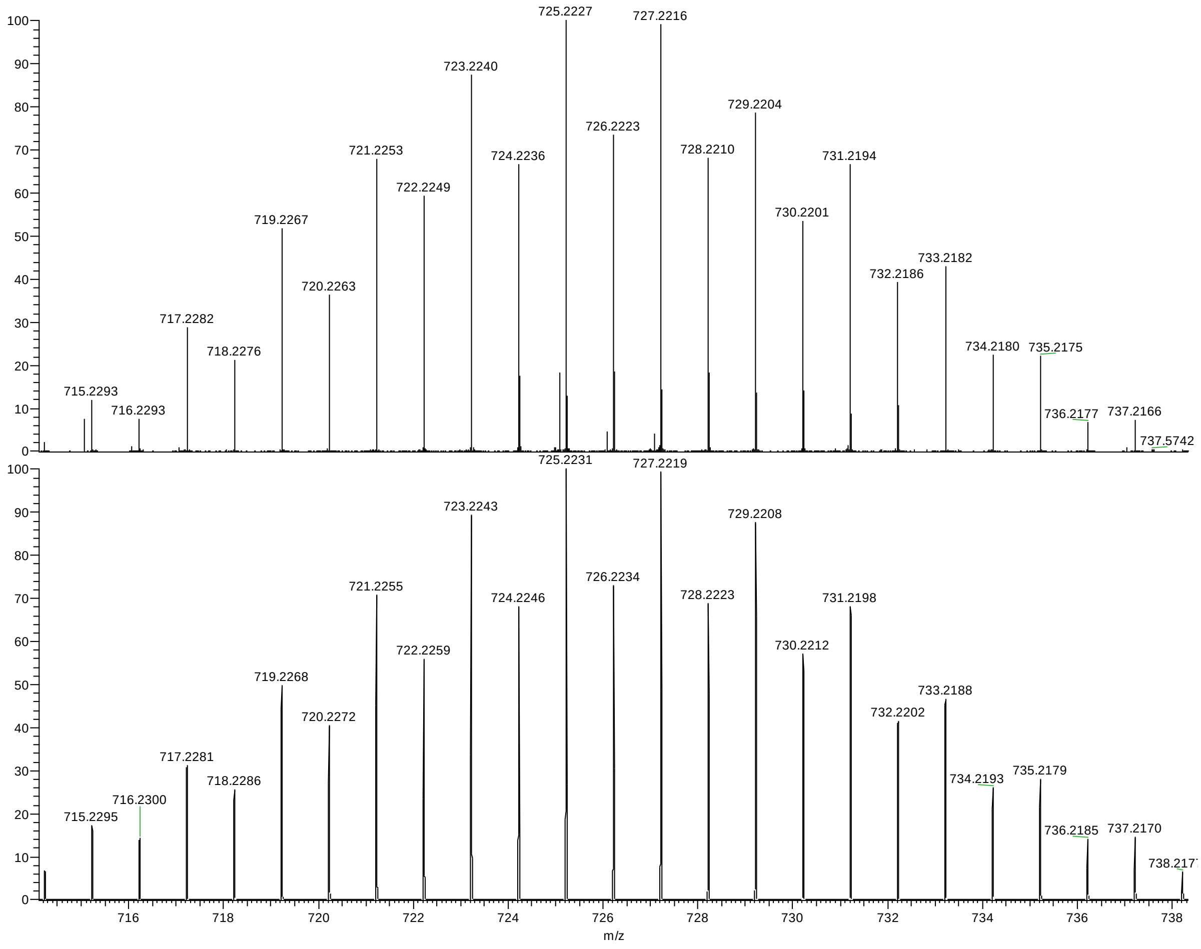

Supplementary Figure 17. ESI(–) mass peak of $(Ge_{10})^-$. Measured (top) vs. calculated (bottom) spectrum.

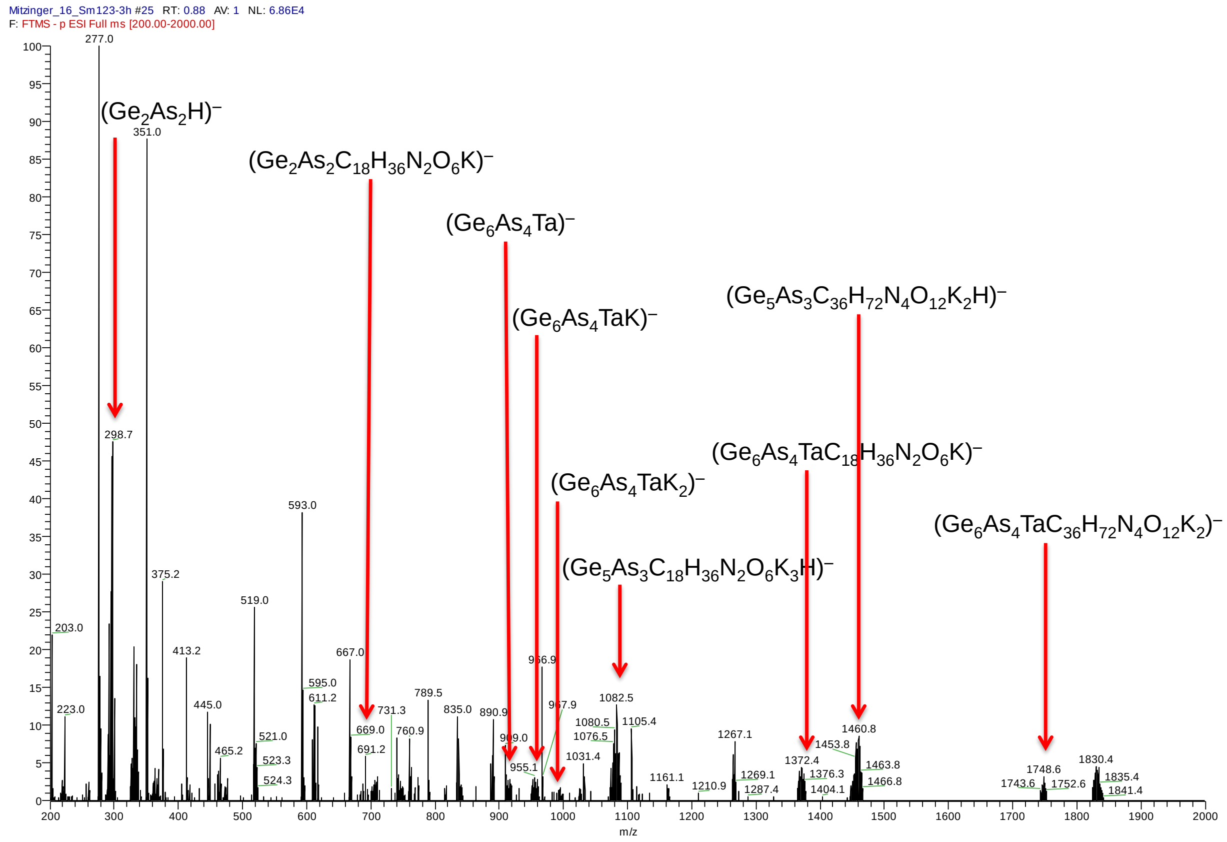

Supplementary Figure 18. ESI-MS(–) overview spectrum of the reaction mixture after 3h of extraction time of the precursor phase “KGeAs:Ta” (precursor phase 1, prepared in a Ta ampoule) in *en* in the presence of [2.2.2]crypt, shown between 200 and 2000 m/z.

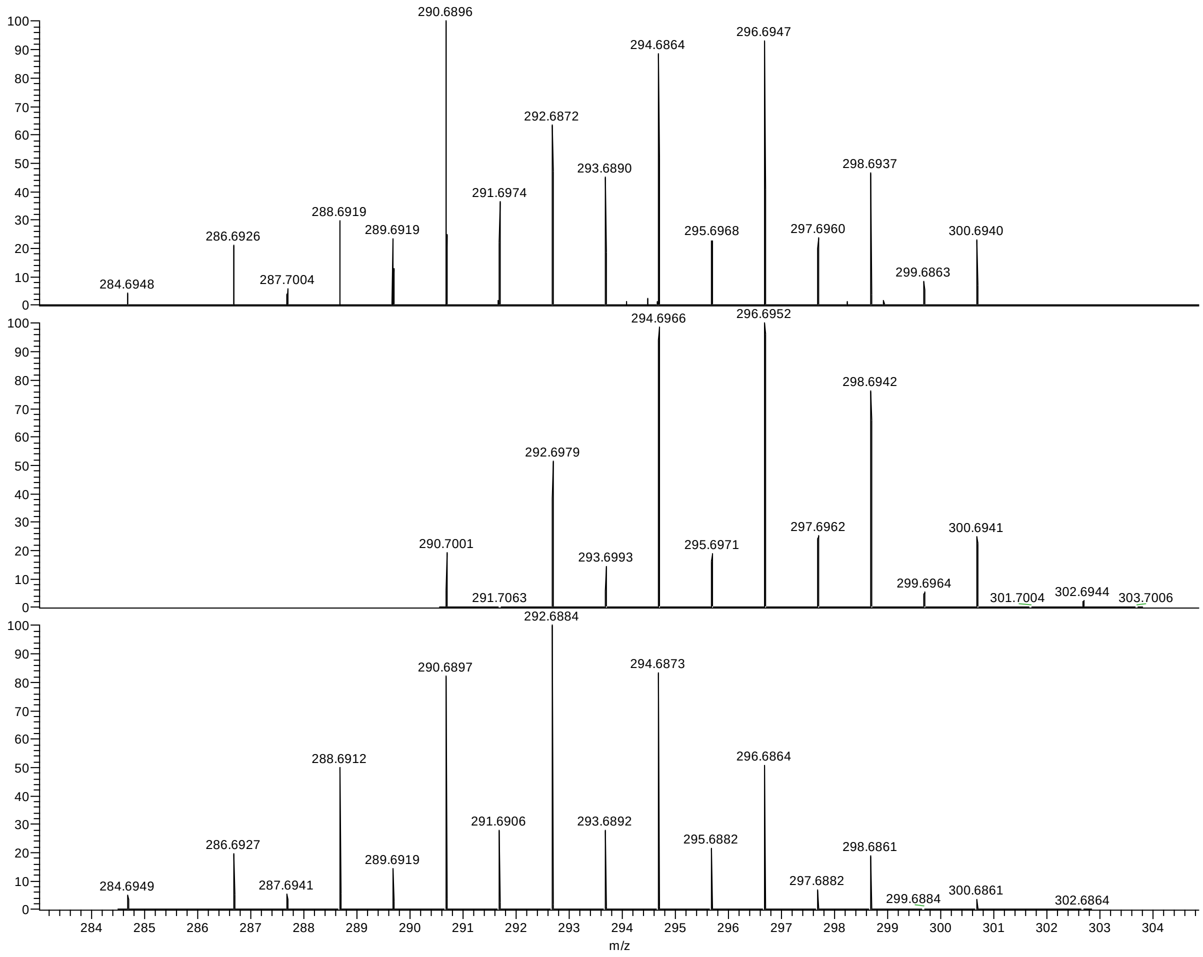

Supplementary Figure 19. ESI(–) mass peak of $(Ge_2As_2H)^-$ and $(Ge_3As)^-$. Measured (top) vs. calculated (center, bottom) spectra.

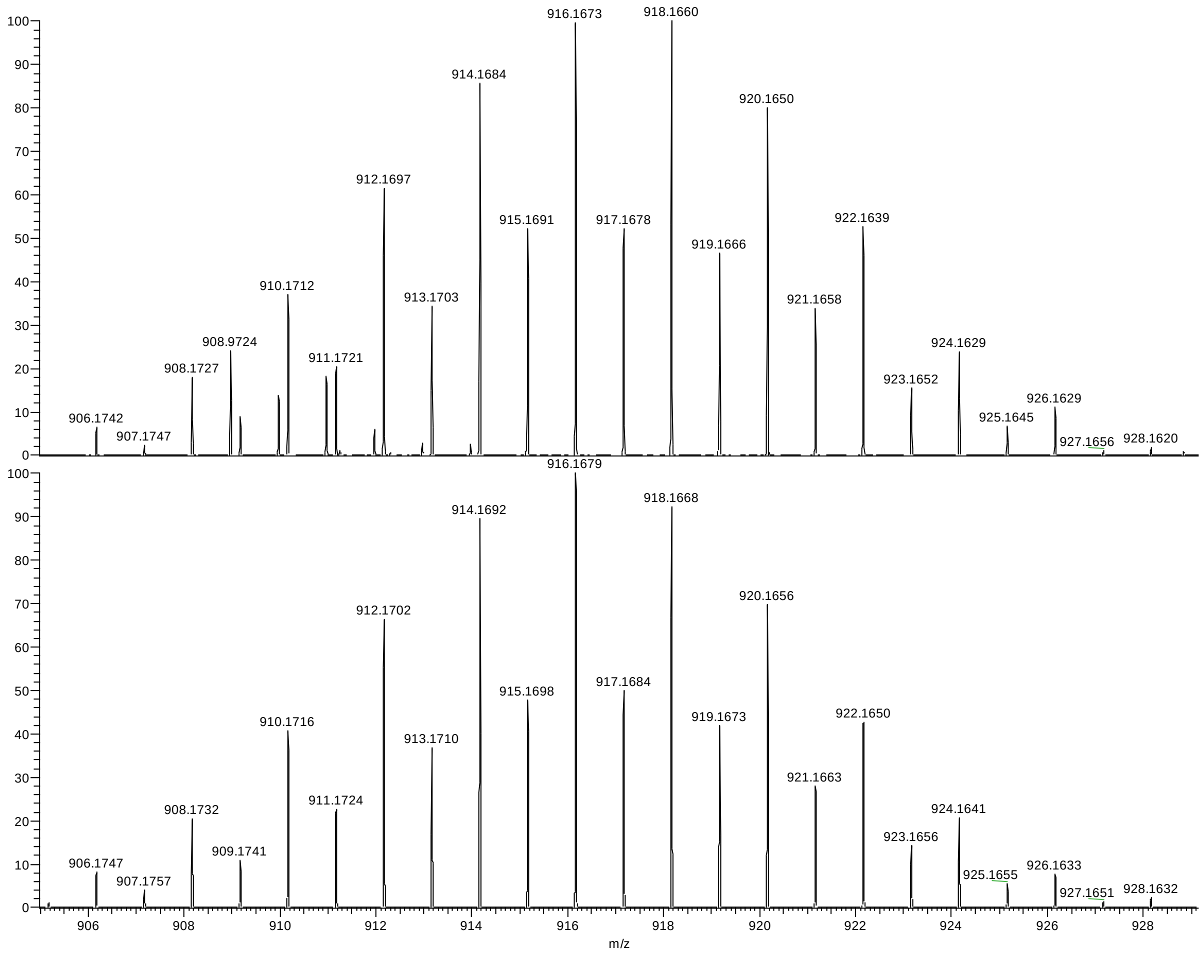

Supplementary Figure 20. ESI(–) mass peak of $(Ge_6As_4Ta)^-$. Measured (top) vs. calculated (bottom) spectrum.

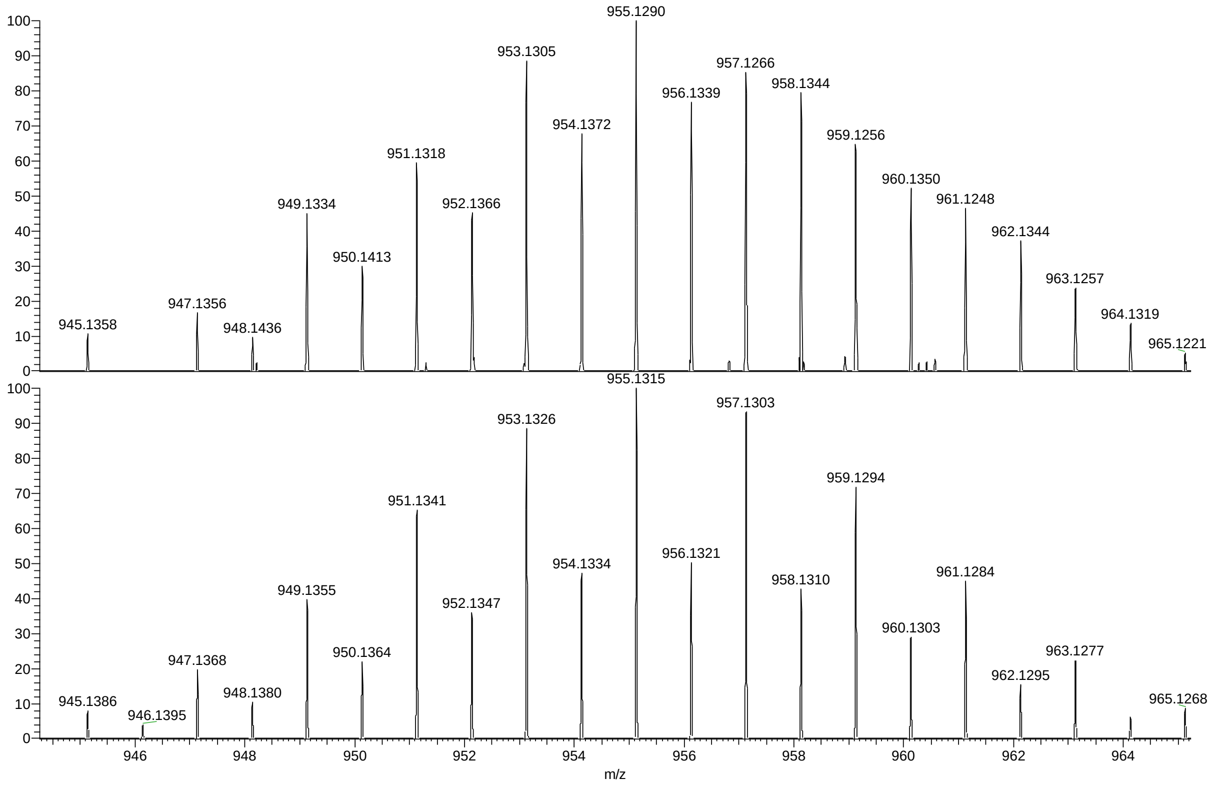

Supplementary Figure 21. ESI(–) mass peak of $(Ge_6As_4TaK)^-$. Measured (top) vs. calculated (bottom) spectrum.

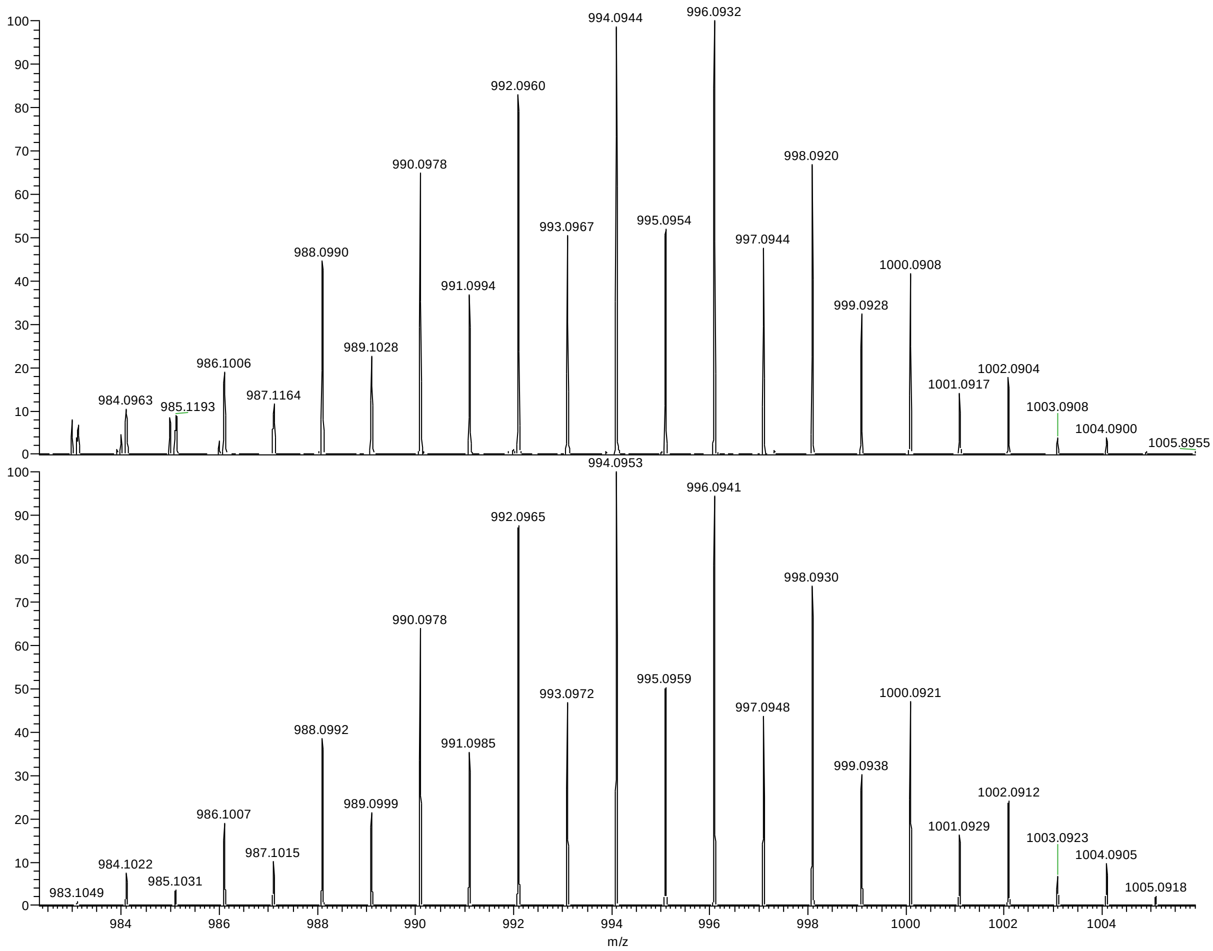

Supplementary Figure 22. ESI(–) mass peak of $(Ge_6As_4TaK_2)^-$. Measured (top) vs. calculated (bottom) spectrum.

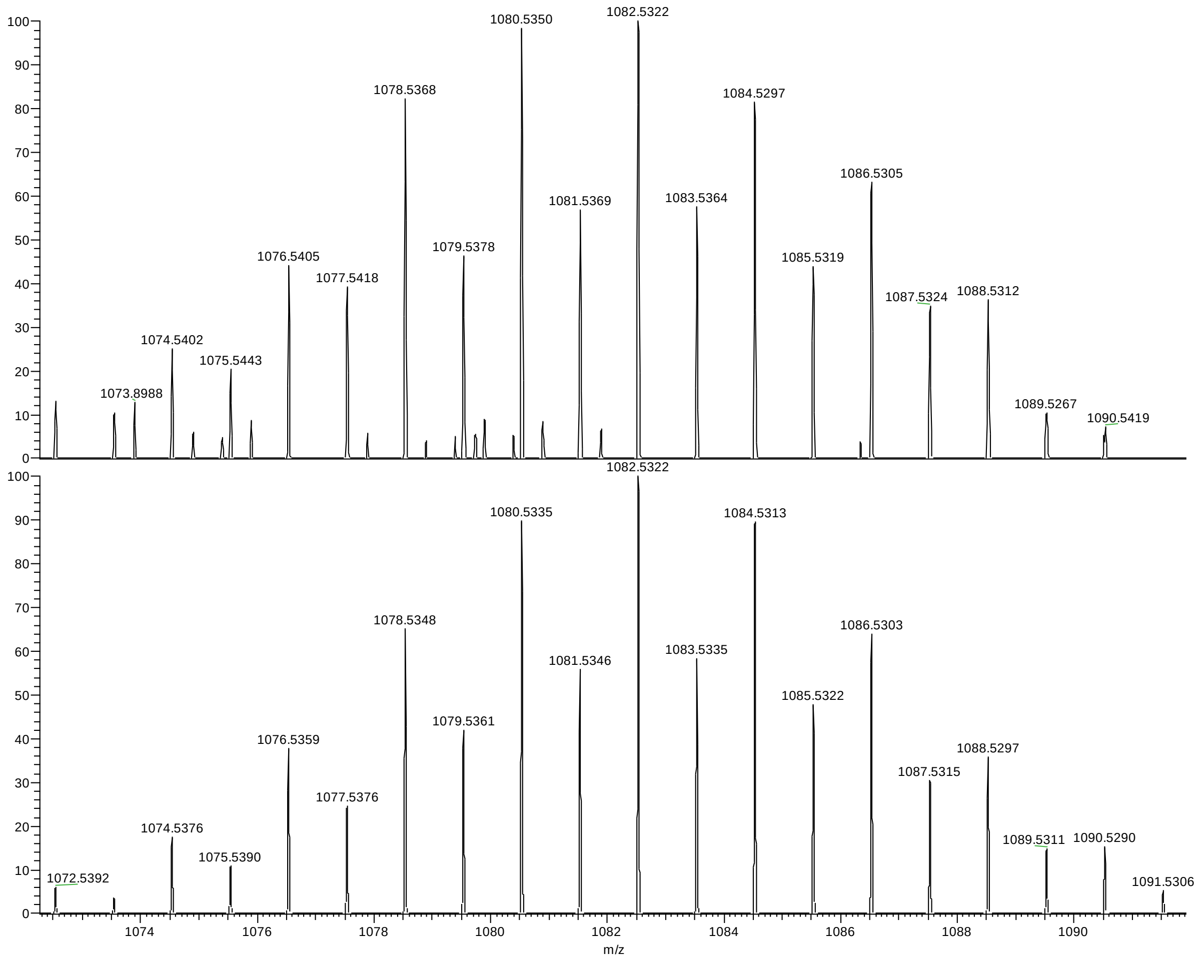

Supplementary Figure 23. ESI(–) mass peak of $(Ge_5As_3C_{18}H_{36}N_2O_6K_3)^-$. Measured (top) vs. calculated (bottom) spectrum.

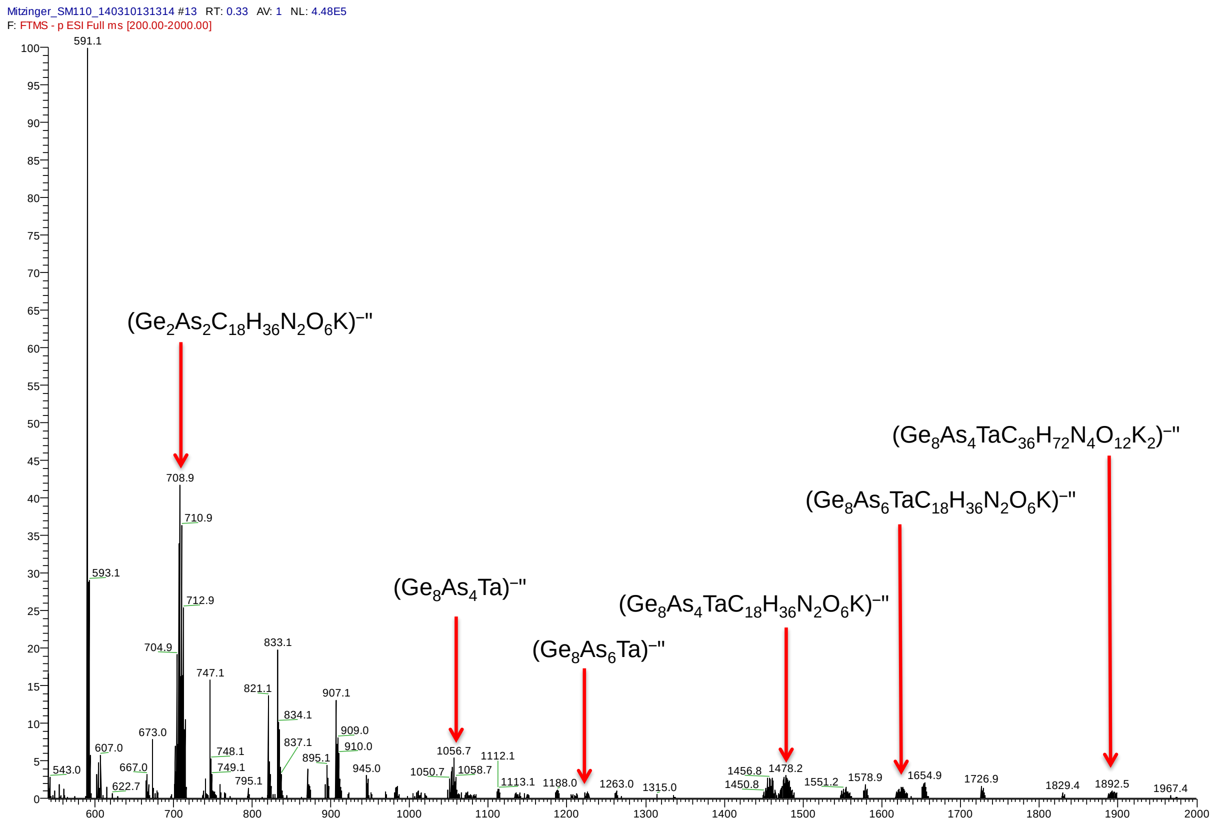

Supplementary Figure 24. ESI-MS(–) overview spectrum of a solution of **4** in DMF/*en*, shown between 625 and 2000 m/z.

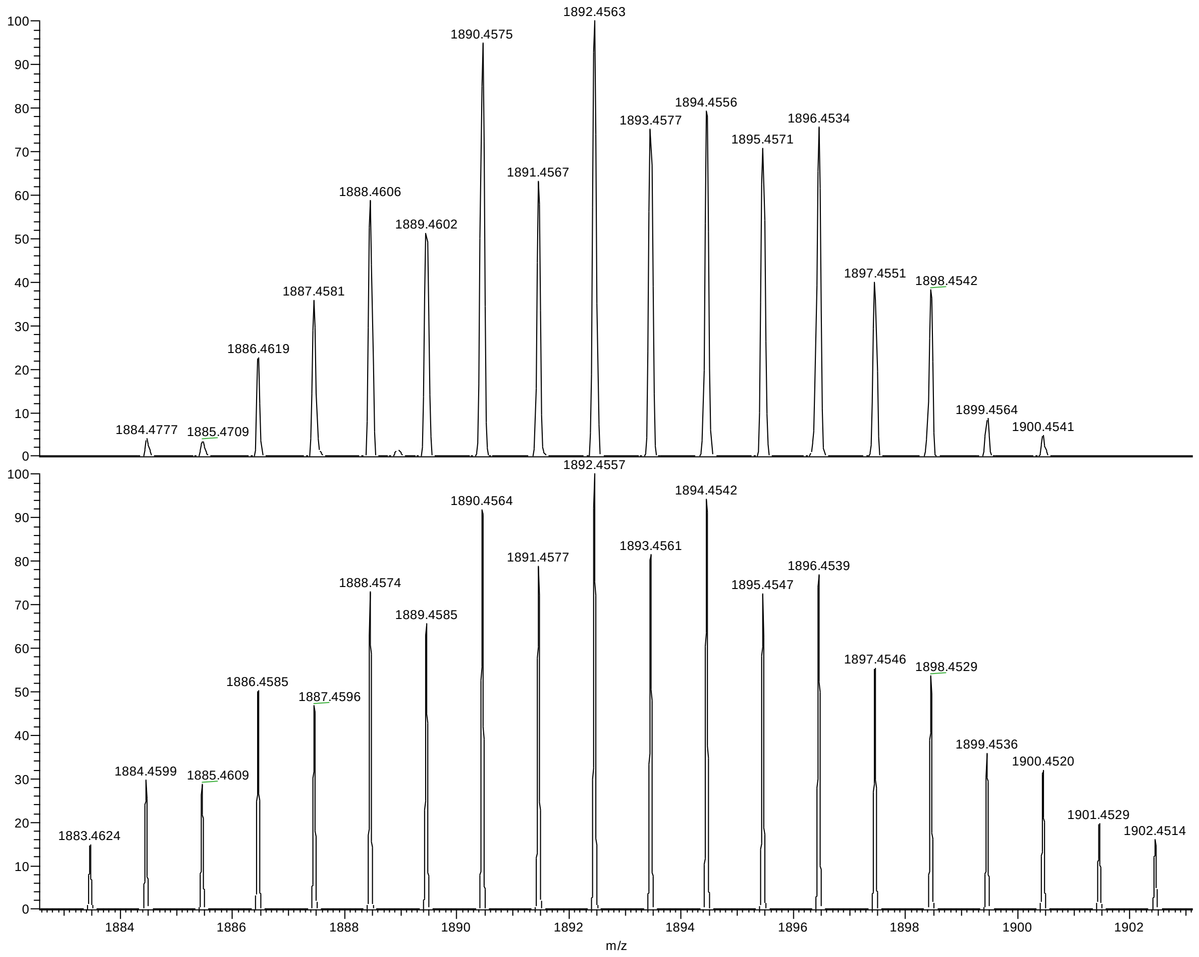

Supplementary Figure 25. ESI(–) mass peak of $(TaGe_8As_4C_{36}H_{72}N_4O_{12}K_2)^-$. Measured (top) vs. calculated (bottom) spectrum.

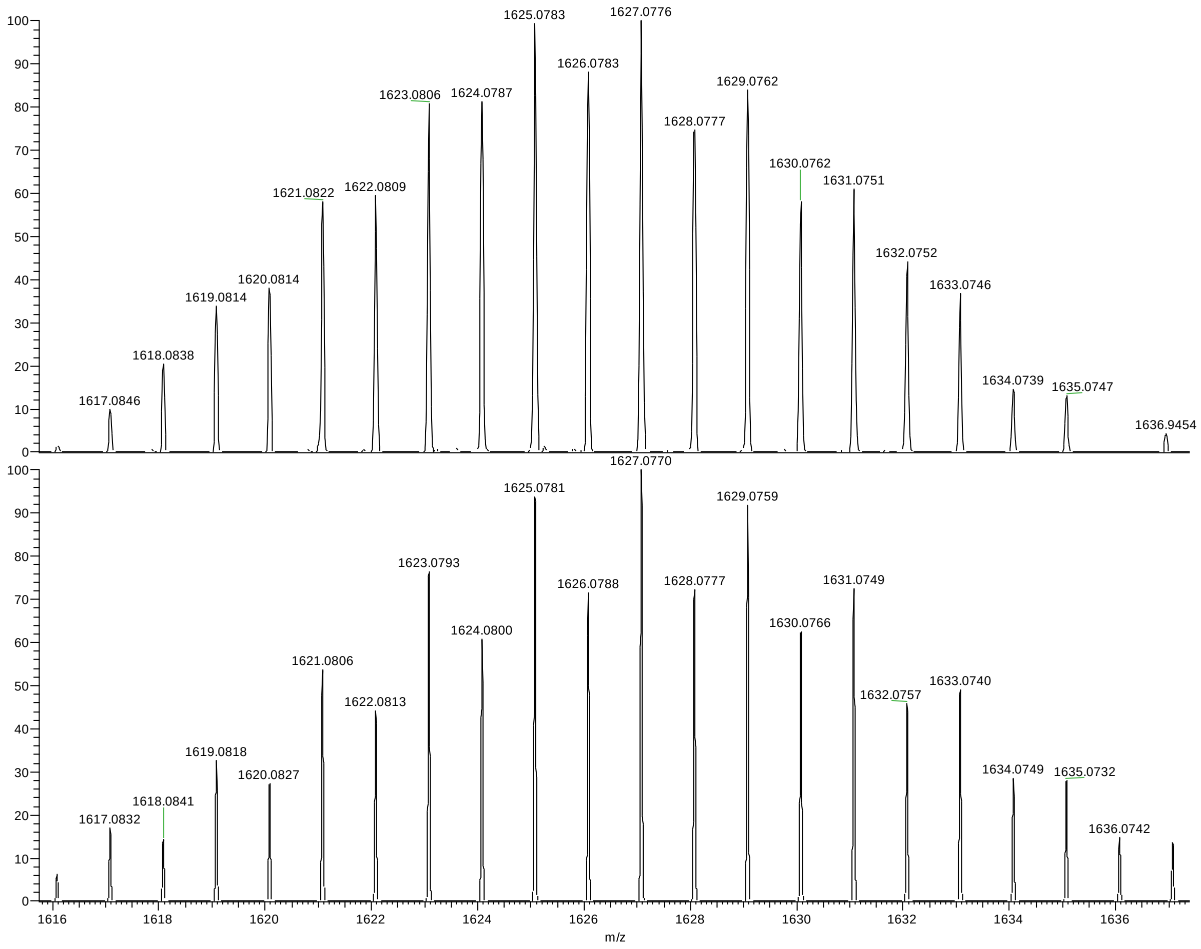

Supplementary Figure 26. ESI(–) mass peak of $(TaGe_8As_6C_{18}H_{36}N_2O_6K)^-$. Measured (top) vs. calculated (bottom) spectrum.

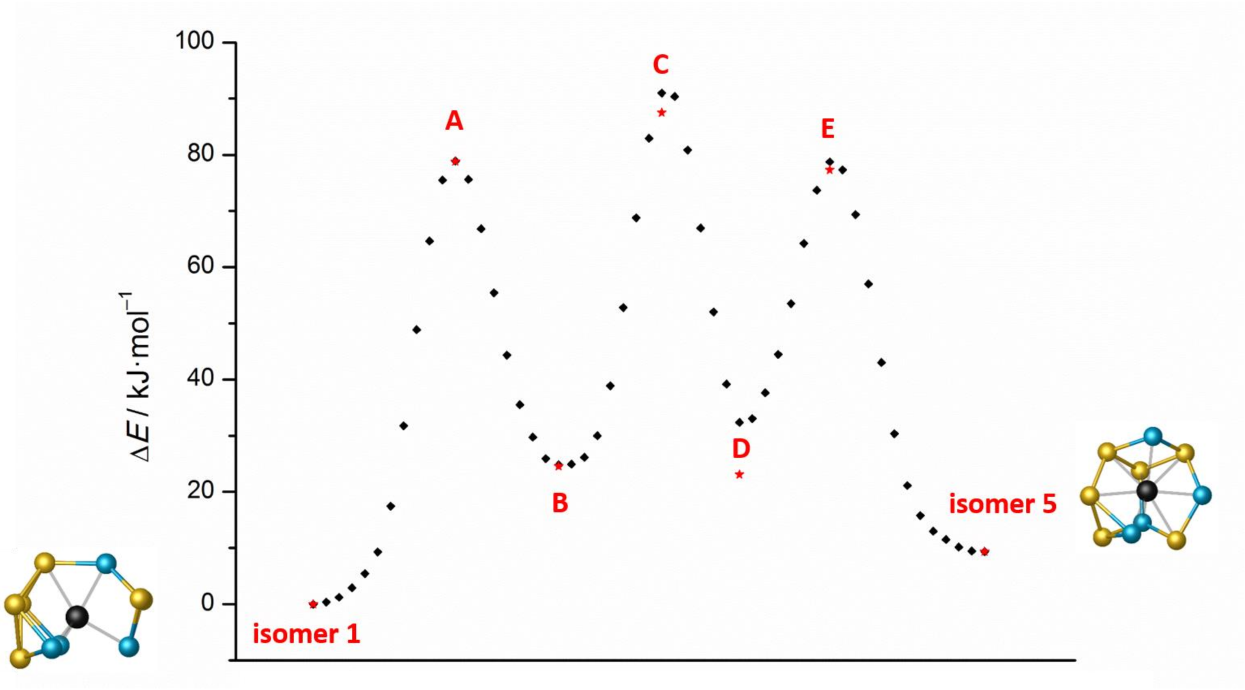

Supplementary Figure 27. Stepwise re-arrangements of the anion of **3**, namely of isomer 1 into isomer 5 (see Figure 5a in the main article). Maxima along the pathways are labeled **A**, **C**, **E**, minima are labeled **B** and **D**. Black points result from the path optimization (PO),[1] the red asterisks from the subsequent individual optimization (IO) of maxima and minima. Δ*E* values with and without this subsequent individual optimization are also listed in Supplementary Table 10, columns "T = 0 K".

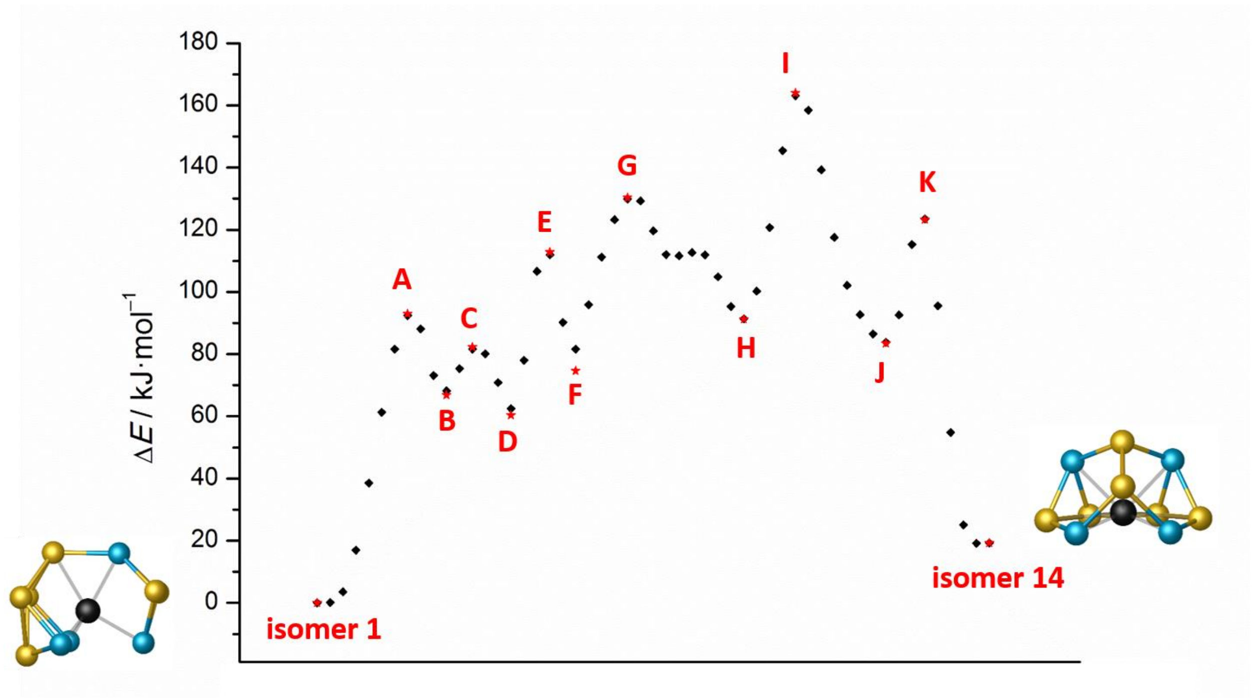

Supplementary Figure 28. Stepwise re-arrangements of the anion of **3**, namely of isomer 1 into isomer 14 (see Figure 5b in the main article). Maxima along the pathways are labeled **A**, **C**, **E**, **G**, **I, K**, minima are labeled **B**, **D**, **F**, **H, J**. Black points result from the path optimization (PO),[1] the red asterisks from the subsequent individual optimization (IO) of maxima and minima. ΔE values with and without this subsequent individual optimization are also listed in Supplementary Table 11, columns "T = 0 K".

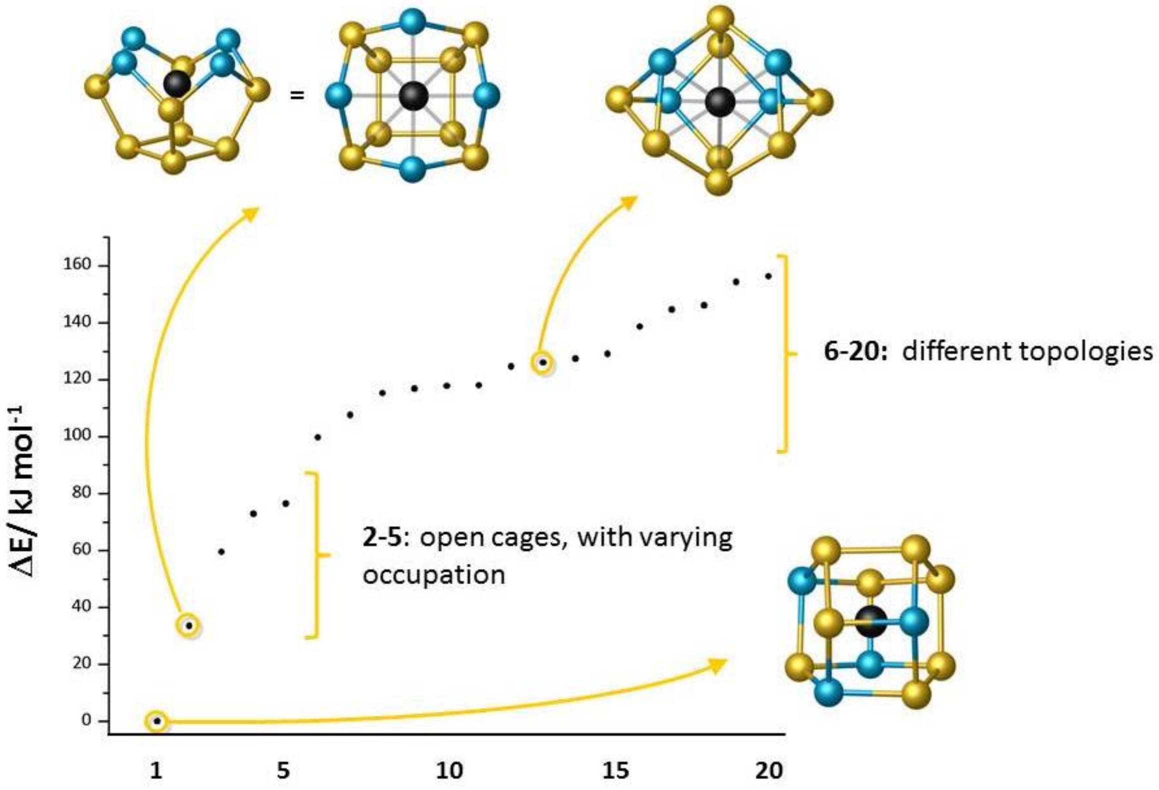

Supplementary Figure 29. Minimum structures for $[Ta@Ge_8As_4]^{3-}$ from GA-RP calculations,[2,3] carried out with the program system TURBOMOLE.[4]

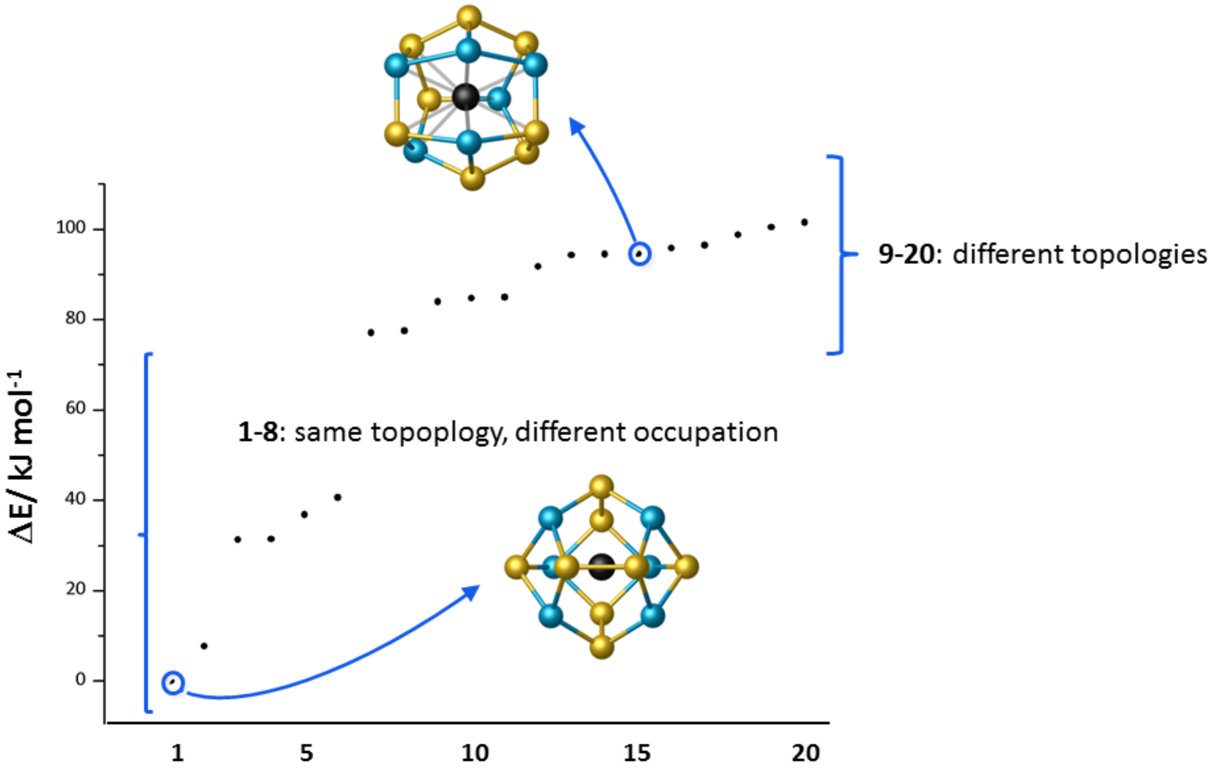

Supplementary Figure 30. Minimum structures for $[Ta@Ge_8As_6]^{3-}$ from GA-RP calculations,[2,3] carried out with the program system TURBOMOLE.[4]

Supplementary Tables

Supplementary Table 1. EDX analysis results of the solid products upon melting a K:Ge:As mixture (1:1:1) in a Ta ampoule (precursor phase 1), or in a silica glass ampoule (precursor phase 2).

Element	k-ratio	ZAF	Atom%	Atomic ratio observed (calc)	Element wt %	wt % Err. (1-sigma)
**Precursor phase 1**						
K-K	0.1808	1.210	36.44	1.24 (1)	21.88	+/- 0.19
Ge-K	0.2704	1.260	30.56	1.04 (1)	34.07	+/- 0.33
As-K	0.3245	1.037	29.26	1.00 (1)	33.67	+/- 1.00
Ta-M	0.0410	2.531	3.73	0.08	10.38	+/- 0.37
Total			100	3.28 (3)	100	
**Precursor phase 2**						
K-K	0.2251	1.169	40.22	1.42 (1)	15.61	+/- 0.47
Ge-K	0.3616	1.056	31.44	1.11 (1)	61.24	+/- 2.99
As-K	0.3352	1.060	28.34	1.00 (1)	23.15	+/- 3.01
Total			100	3.54 (3)	100	

Supplementary Table 2. Energy dispersive X-ray (EDX) analysis of single crystals of compounds **1**, **2**, **3**, and **4** (K, Ge, As, Ta).

Element	k-ratio	ZAF	Atom%	Atomic ratio observed (calc)	Element wt %	wt % Err. (1-sigma)
**[K([2.2.2]crypt)]$_2$(Ge$_2$As$_2$)·en (1)**						
K-K	0.1519	1.056	26.48	1.50 (2)	16.04	+/- 0.42
Ge-K	0.4166	1.035	38.34	2.17 (2)	43.12	+/- 4.29
As-K	0.3918	1.042	35.18	2.00 (2)	40.84	+/- 3.43
Total			100	5.67 (6)	100	
**[K([2.2.2]crypt)]$_2$(Ge$_7$As$_2$) (2)**						
K-K	0.1318	1.247	25.72	2.58 (2)	15.61	+/- 0.47
Ge-K	0.5929	1.019	54.37	5.45 (7)	61.24	+/- 2.99
As-K	0.2232	1.018	19.91	2.00 (2)	23.15	+/- 3.01
Total			100	10.03 (11)	100	
**[K([2.2.2]crypt)]$_3$[Ta@Ge$_6$As$_4$]·2tol (3)**						
K-K	0.1595	1.247	35.90	7.02 (3)	19.89	+/- 0.34
Ge-K	0.3518	1.019	34.85	6.81 (6)	35.85	+/- 1.84
As-K	0.2135	1.018	20.47	4.00 (4)	21.73	+/- 1.86
Ta-M	0.1997	1.128	8.78	1.72 (1)	22.53	+/- 1.89
Total			100	19.55 (14)		
**[K([2.2.2]crypt)]$_5$[K([2.2.2]crypt)(en)][Ta@Ge$_8$As$_4$]$_{1.21}$[Ta@Ge$_8$As$_6$]$_{0.79}$·en (4)**						
K-K	0.0813	1.258	17.87	3.05 (3)	9.73	+/- 0.36
Ge-K	0.3848	1.001	47.65	6.19 (8)	46.40	+/- 2.42
As-K	0.3205	1.001	28.53	5.00 (5)	28.86	+/- 2.72
Ta-M	0.0759	2.528	5.96	1.24 (1)	15.01	+/- 0.99
Total			100	15.48 (17)	100	

Supplementary Table 3. Crystallographic data for the X-ray structure analyses of **1** - **4**.

	**1**	**2**	**3**	**4**
**General data**				
Empirical formula	$C_{38}H_{80}N_6O_{12}K_2$-Ge_2As_2	$C_{36}H_{72}N_4O_{12}K_2$-Ge_7As_2	$C_{68}H_{124}N_6O_{18}K_3$-$Ge_6As_4Ta$	$C_{56}H_{116}N_8O_{18}K_3$-$As_{4.79}Ge_8Ta$
Formula weight [$g·mol^{-1}$]	1186.30	1489.14	2347.19	2427.40
Crystal color and shape	orange block	orange block	red plate	black block
Crystal size [mm^3]	0.34×0.28×0.19	0.32×0.30×0.20	0.50×0.33×0.12	0.30×0.09×0.08
**Data collection**				
Crystal system	triclinic	trigonal	monoclinic	triclinic
Space group	$P1$	$P\bar{3}c1$	$P2_1/n$	$P\bar{1}$
a [Å]	10.9738(4)	11.8653(3)	13.7543(2)	16.6082(5)
b [Å]	11.9313(4)	11.8653(3)	28.6077(5)	22.6960(8)
c [Å]	12.6146(6)	22.3848(9)	22.9845(4)	23.9068(8)
α [°]	118.021(3)			94.960(3)
β [°]	108.361(4)		92.489(1)	94.036(3)
γ [°]	96.476(3)			91.402(3)
V [$Å^3$]	1315.07(10)	2729.2(2)	9035.4(3)	8951.2(5)
Z, ρ_{calc} [$g·cm^{-3}$]	1	2	4	4
**Refinement**				
μ ($Mo_{K\alpha}$) [mm^{-1}]	2.608	5.212	4.829	5.819
absorption correction type	Gaussian	Gaussian	Gaussian	Gaussian
2θ range [°]	2.68-26.82	1.82-26.76	1.42-26.80	2.55-25.00
total reflns	23683	3581	148425	64236
unique reflns [R_{int}]	10275 [0.065]	1942 [0.034]	19185 [0.070]	31253 [0.057]
obs. reflns [$I > 2\sigma(I)$]	9372	783	13496	16662
parameters	579	119	982	1597
R_1 [$I > 2\sigma(I)$]	0.0454	0.0521	0.0346	0.0735
wR_2 (all data)	0.1189	0.1048	0.0795	0.1973
GooF (all data)	1.076	0.958	0.884	0.991
max peak/hole, [$e·Å^{-3}$]	1.17/ -0.80	0.65/ -0.45	1.20/ -0.62	3.38/ -1.72

Supplementary Table 4. Interatomic distances (in Å) in the $(Ge_2As_2)^{2-}$ anion in **1** (Supplementary Figure 3a). Angles are 58.30(3) - 62.71(3)°.

Atom numbers		Atom numbers	
1–2	2.459(1)	2–4	2.568(1)
1–3	2.503(1)	3–4	2.517(1)
1–4	2.477(1)		
2–3	2.572(1)	Average	**2.516(1)**

Supplementary Table 5. Interatomic distances (in Å) in the $(Ge_7As_2)^{2-}$ anion in **2** (Supplementary Figure 5a).

Atom numbers		Atom numbers	
1–2	2.416(3)	2–4	2.612(5)
1–3	2.542(5)	3–4	2.409(7)
1–4	2.729(3)	3–5	2.708(9)
1–5	2.696(5)	4–5	2.643(8)
		Average	**2.594**

Supplementary Table 6. Interatomic distances (in Å) in the two isomers of the $[Ta@Ge_6As_4]^{3-}$ anion in **3** (Supplementary Figure 7a and 7b).

**Isomer 1**					
Ta1 – As1	2.505(1)	Ta1 ··Ge10	3.1222(7)	Ge4–Ge3	2.519(1)
Ta1–Ge3	2.719(1)	As1–Ge6	2.525(2)	Ge3–As7	2.627(1)
Ta1–As5	2.496(1)	As5–Ge6	2.522(1)	As7–Ge8	2.519(1)
Ta1–As7	2.6229(7)	Ge2–Ge6	2.733(1)	As7–Ge10	2.505(1)
Ta1 ··· Ge8	3.1089(8)	Ge4 – Ge6	2.761(1)	As9 – Ge8	2.4908(8)
Ta1 – As9	2.5413(6)	Ge2 – Ge3	2.543(1)	As9 – Ge10	2.4770(7)
**Isomer 2**					
Ta1A–As1A	2.46(1)	As1A–Ge6A	2.53(1)	Ge7A–Ge8A	2.600(8)
Ta1A–As3A	2.52(1)	As5A–Ge6A	2.44(1)	Ge7A–Ge9B	2.53(1)
Ta1A–As5A	2.494(8)	Ge6A–Ge2A	2.67(1)	Ge7A–As9A	2.594(7)
Ta1A–Ge7A	2.746(7)	Ge6A–Ge4A	2.56(1)	Ge8A–As9A	2.705(7)
Ta1A–Ge8A	2.699(6)	As3A–Ge2A	2.47(1)	Ge9B–As9A	2.349(9)
Ta1A–As9A	2.906(3)	As3A–Ge4A	2.47(1)	Ge9B–Ge8A	2.53(1)

Supplementary Table 7. Interatomic distances (in Å) in the $[Ta@Ge_8As_4]^{3-}$ anion in **4** (Supplementary Figure 10a) and interatomic distances (in Å) in the $[Ta@Ge_8As_6]^{3-}$ anion in **4** (Supplementary Figure 10b).

**$[Ta@Ge_8As_4]^{3-}$ Anion in 4**					
Ta–1	2.842(1)	Ta–6	2.790(1)	Ta–11	2.824(2)
Ta–2	2.708(1)	Ta–7	2.790(1))	Ta–12	2.795(2)
Ta–3	2.727(1)	Ta–8	2.731(1)		
Ta–4	2.759(2)	Ta–9	2.742(2)	average	**2.763**
Ta–5	2.706(1)	Ta–10	2.739(1)		
1–2	2.571(2)	3–11	2.572(2)	7–8	2.533(2)
1–5	2.574(2)	4–5	2.526(2)	8–9	2.600(2)
1–6	2.491(2)	4–12	2.530(2)	9–10	2.541(2)
2–3	2.515(2)	5–10	2.591(2)	11–12	2.527(2)
2–7	2.639(2)	6–7	2.509(2)		
3–4	2.678(2)	6–10	2.482(2)	average	**2.555**
**$[Ta@Ge_8As_6]^{3-}$ Anion in 4**					
Ta–Ge1	2.842(1)	Ta–Ge8	2.731(1)	Ta–As1d	2.729(12
Ta–As2	2.708(1)	Ta–As11	2.824(1)	Ta–As1e	3.037(11)
Ta–Ge3	2.727(1)	Ta–Ge1a	3.193(12)	Ta–Ge1f	3.037(15)
Ta–As6	2.790(1)	Ta–Ge1b	2.906(12)	Ta–Ge1g	3.125(14)
Ta–Ge7	2.790(1)	Ta–As1c	3.097(15)		
			average	Ta–Ge/As	**2.895**
Ge1–As2	2.571(2)	As6–Ge1a	2.816(12)	Ge1a–As1e	2.49(2)
Ge1–Ge1b	2.108(13)	Ge7–Ge8	2.533(2)	Ge1b–As1d	2.48(2)
Ge1–As6	2.491(2)	Ge8–As11	2.542(2)	Ge1b–As1e	2.35(2)
As2–Ge3	2.515(2)	Ge8–As1c	2.32(1)	As1c–Ge1f	2.47(2)
As2–Ge7	2.639(2)	As6–Ge7	2.509(2)	As1d–Ge1g	2.50(2)
Ge3–As1d	2.064(11)	As11–Ge1f	2.33(1)	As1e–Ge1g	2.48(2)
Ge3–As11	2.572(2)	As6–Ge1a	2.816(12)	Ge1g–Ge1f	2.49(2)
As6–Ge7	2.509(2)	Ge1a–As1c	2.60(2)		
			average	Ge/As–Ge/As	**2.472**

Supplementary Table 8. Comparison of Δ*E* values (in kJ·mol^{-1}) at 0 K for the species specified by red asterisks ("IO") in Supplementary Figure 27, calculated with dhf-TZVP[5,6] basis using different functionals: TPSS,[7] BP86,[8,9] PBE,[10] and TPSSh.[11] The barrier heights for different functionals were calculated from the respective data. The numbers in parentheses are the changes compared to the values obtained with the functional TPSS.

**Δ*E* / kJ·mol^{-1}**				
	**TPSS**	**BP86**	**PBE**	**TPSSh**
**isomer 1**	0	0	0	0
**A**	78.8	76.8	77.5	83.5
**B**	24.6	25.9	25.0	26.2
**C**	87.5	83.9	85.3	90.0
**D**	23.1	21.1	22.9	24.1
**E**	77.2	71.3	73.2	77.4
**isomer 5**	9.4	7.6	7.3	7.5
**Barrier heights / kJ·mol^{-1}**				
	**TPSS**	**BP86**	**PBE**	**TPSSh**
**1→A**	78.8	76.8 (–2.0)	77.5 (–1.3)	83.5 (+4.7)
**B→C**	62.9	58.1 (–4.8)	60.4 (–2.5)	63.8 (+0.9)
**D→E**	54.2	50.2 (–4.0)	50.2 (–4.0)	53.3 (–0.9)

Supplementary Table 9. Comparison of Δ*E* values (in kJ·mol^{-1}) at 0 K for the species specified by red asterisks ("IO") in Supplementary Figure 28, calculated with dhf-TZVP[5,6] basis using different functionals: TPSS,[7] BP86,[8,9] PBE,[10] and TPSSh.[11] The barrier heights for different functionals were calculated from the respective data. The numbers in parentheses are the changes compared to the values obtained with the functional TPSS.

**Δ*E* / kJ·mol^{-1}**				
	**TPSS**	**BP86**	**PBE**	**TPSSh**
**isomer 1**	0	0.0	0.0	0.0
**A**	92.9	88.9	89.3	97.5
**B**	66.8	71.0	68.2	68.0
**C**	82.2	86.2	83.4	84.3
**D**	60.4	64.4	61.7	61.1
**E**	112.7	108.9	109.2	115.4
**F**	74.6	74.8	73.3	77.7
**G**	130.3	129.6	130.0	135.1
**H**	91.2	84.7	87.1	90.7
**I**	163.9	156.0	158.0	170.8
**J**	83.5	84.2	82.9	86.3
**K**	123.3	115.0	118.2	127.0
**isomer 14**	19.3	18.9	17.9	17.9
**Barrier heights / kJ·mol^{-1}**				
	**TPSS**	**BP86**	**PBE**	**TPSSh**
**1→A**	92.9	88.9 (–4.0)	89.3 (–3.6)	97.5 (+4.6)
**B→C**	15.4	15.2 (–0.2)	15.1 (–0.3)	16.3 (+0.9)
**D→E**	52.4	44.5 (–7.9)	47.6 (–4.8)	54.4 (+2.0)
**F→G**	55.6	54.8 (–0.8)	56.7 (+1.1)	57.4 (+1.8)
**H→I**	72.7	71.4 (–1.3)	71.0 (–1.7)	80.1 (+7.4)
**J→K**	39.8	30.7 (–9.1)	35.3 (–4.5)	40.6 (+0.8)

Supplementary Table 10. Comparison of ΔE values (in kJ·mol^{-1}) at 0 K for the species specified in Supplementary Fig. 27 resulting directly from the path optimization (PO,[1] black points in Supplementary Fig. 27) and with subsequent individual geometry optimization (IO, red asterisks in Supplementary Fig. 27) and with ΔG values at different temperatures, up to 900 K for the IO species, calculated from partition sums within the standard harmonic oscillator approximation for molecules in the gas phase.[12] The vibrational frequencies were used non-scaled. All values were calculated at dhf-TZVP/TPSS level.[5-7] The barrier heights for different functionals were calculated from the respective data. The numbers in parentheses are the changes compared to the values obtained for the IO species at 0K (column "T = 0 K, IO").

	**ΔE / kJ·mol^{-1}**		**ΔG / kJ·mol^{-1}**			
	**T = 0 K, PO**	**T = 0 K, IO**	**T = 298 K**	**T = 500 K**	**T = 700 K**	**T = 900 K**
**isomer 1**	0	0	0	0	0	0
**A**	78.9	78.8	79.7	82.4	85.9	89.8
**B**	24.8	24.6	24.9	25.2	25.4	25.7
**C**	91.0	87.5	89.3	92.7	96.8	101.4
**D**	32.4	23.1	23.9	24.4	24.8	25.3
**E**	78.3	77.2	81.2	86.2	91.7	97.8
**isomer 5**	9.4	9.4	11.9	13.6	15.4	17.1
**Barrier heights / kJ·mol^{-1}**						
	**T = 0 K, PO**	**T = 0 K, IO**	**T = 298 K**	**T = 500 K**	**T = 700 K**	**T = 900 K**
**1→A**	78.9	78.8	79.7 (+0.9)	82.4 (+3.6)	85.9 (+7.1)	89.8 (+11.0)
**B→C**	66.2	62.9	64.4 (+1.5)	67.5 (+4.6)	71.3 (+8.4)	75.7 (+12.8)
**D→E**	46.4	54.2	57.4 (+3.2)	61.8 (+7.6)	66.9 (+12.7)	72.5 (+18.3)

Supplementary Table 11. Comparison of Δ*E* values (in kJ·mol^{-1}) at 0 K for the species specified in Supplementary Figure 28 resulting directly from the path optimization (PO,[1] black points in Supplementary Figure 28) and with subsequent individual geometry optimization (IO, red asterisks in Supplementary Figure 28) and with Δ*G* values at different temperatures, up to 900 K for the IO species, calculated from partition sums within the standard harmonic oscillator approximation for molecules in the gas phase.[12] The vibrational frequencies were used non-scaled. All values were calculated at dhf-TZVP/TPSS level.[5-7] The barrier heights for different functionals were calculated from the respective data. The numbers in parentheses are the changes compared to the values obtained for the IO species at 0K (column "T = 0 K, IO").

	**Δ*E* / kJ·mol^{-1}**		**Δ*G* / kJ·mol^{-1}**			
	**T = 0 K, PO**	**T = 0 K, IO**	**T = 298 K**	**T = 500 K**	**T = 700 K**	**T = 900 K**
**isomer 1**	0	0	0.0	0.0	0.0	0.0
**A**	92.4	92.9	92.8	95.0	97.8	101.1
**B**	68.2	66.8	64.5	63.0	61.5	60.0
**C**	81.7	82.2	82.4	84.7	87.6	91.1
**D**	62.4	60.4	58.7	57.8	56.7	55.7
**E**	112.0	112.8	111.4	112.7	114.7	117.1
**F**	81.6	74.6	74.8	75.1	75.3	75.5
**G**	129.9	130.3	127.5	127.9	129.0	130.5
**H**	91.4	91.2	90.7	90.4	90.1	89.8
**I**	163.1	163.9	163.1	164.8	167.1	170.0
**J**	83.8	83.5	82.3	81.7	81.0	80.3
**K**	123.5	123.5	122.5	124.2	126.6	129.4
**isomer 14**	19.3	19.3	24.0	27.3	30.5	33.7
**Barrier heights / kJ·mol^{-1}**						
	**T = 0 K, PO**	**T = 0 K, IO**	**T = 298 K**	**T = 500 K**	**T = 700 K**	**T = 900 K**
**1→A**	92.4	92.9	92.8 (–0.1)	95.0 (+2.1)	97.8 (+4.9)	101.1 (+8.2)
**B→C**	13.5	15.4	17.9 (+2.5)	21.7 (+6.3)	26.2 (+10.8)	31.1 (+15.7)
**D→E**	49.5	52.4	52.6 (+0.2)	54.9 (+2.5)	57.9 (+5.5)	61.4 (+9.0)
**F→G**	48.3	55.6	52.6 (–3.0)	52.8 (–2.8)	53.7 (–1.9)	55.0 (–0.6)
**H→I**	71.7	72.7	71.4 (–0.3)	74.4 (+1.7)	77.0 (+4.3)	80.2 (+7.5)
**J→K**	39.7	39.8	40.1 (+0.3)	42.5 (+2.7)	45.5 (+5.7)	49.0 (+9.2)

Supplementary Table 12. Reaction energy differences (in kJ·mol^{-1}) for the charge-conserving second and the third step of the mechanism proposed in the manuscript, calculated with basis sets def-SVP[13-15] and functional BP86,[8,9] or with basis set dhf-TZVP[5,6] and functionals BP86, PBE,[10] TPSSh[11] or TPSS.[7] The dielectric constant, *ε*, in COSMO[16] was set to infinity (default) as well as to 13.5, which is the value for 1,2-diaminoethane (*en*), in case of the calculations at level dhf-TZVP/ TPSS.

Basis set	def-SVP	dhf-TZVP				
Functional/basis set	BP86	BP86	PBE	TPSSh	TPSS	
ε	∞	∞	∞	∞	∞	13.5
2nd step :<br>$(Ge_7As_2)^{2-}$ + 2 Ta<br>→ $[Ta@Ge_4As_2]^-$ + $(TaGe_3)^-$	–815.6	–874.5	−883.7	-866.6	–902.9	–917.1
3rd step:<br>$[Ta@Ge_4As_2]^-$ + $(Ge_2As_2)^{2-}$<br>→ $[Ta@Ge_6As_4]^{3-}$	–312.4	–318.8	−333.7	−331.9	–330.6	–300.6

Supplementary References

1 Plessow. P. Reaction Path Optimization without NEB Springs or Interpolation Algorithms. J. Chem. Theory Comput. 9 (3), 1305-1310, (2013).

2 Weigend, F. Extending DFT-based genetic algorithms by atom-to-place re-assignment via perturbation theory: A systematic and unbiased approach to structures of mixed-metallic clusters. J. Chem. Phys. 141, 134103 (2014).

3 Deaven, D. M. & Ho, K. M. Molecular Geometry Optimization with a Genetic Algorithm. Phys. Rev. Lett. 75, 288-291 (1995).

4 TURBOMOLE Version 6.6, © TURBOMOLE GmbH 2014. TURBOMOLE is a development of University of Karlsruhe and Forschungszentrum Karlsruhe 1989 − 2007, TURBOMOLE GmbH since 2007.

5 Weigend, F, & Baldes, A. Segmented contracted basis sets for one- and two-component Dirac-Fock effective core potentials. J. Chem. Phys. 133, 174102 (2010).

6 Figgen, D., Peterson, K. A., Dolg, M. & Stoll, H. Correlation consistent basis sets: the transition metals Hf – Pt. J. Chem. Phys. 130, 164108 (2009).

7 Tao, J., Perdew, J. P., Staroverov, V. N. & Scuseria, G. E. Climbing the density functional ladder: nonempirical meta–generalized gradient approximation designed for molecules and solids. Phys. Rev. Lett. 91, 146401 (2003).

8 Becke, A. D. Density-functional exchange-energy approximation with correct asymptotic behavior. Phys. Rev. A 38, 3098-3100 (1988).

9 Perdew, J. P. Density-functional approximation for the correlation energy of the inhomogeneous electron gas. Phys. Rev. B 33, 8822-8824 (1986).

10 Perdew, J.P., Burke, K. & Ernzerhof, M. Generalized gradient approximation made simple. Phys. Rev. Lett. 77, 3865-3868 (1996).

11 Staroverov, V.N., Scuseria, G.E, Tao & J., Perdew, J.P. Comparative assessment of a new nonempirical density functional: Molecules and hydrogen-bonded complexes. J. Chem. Phys. 119, 12129-12137 (2003).

12 McQuarrie, D. A. & Simon, J.D. Molecular Thermodynamics, University Science Books, ISBN 978-1891389054 (1999).

13 Schäfer, A., Horn, H. & Ahlrichs, R. Fully optimized contracted Gaussian basis sets for atoms Li to Kr. J. Chem. Phys. 97, 2571 (1992).

14 Eichkorn, K., Weigend, F., Treutler, O. & Ahlrichs, R. Auxiliary basis sets for main row atoms and transition metals and their use to approximate coulomb potentials. Theor. Chem. Acc., 97, 119-124, (1997).

15 Andrae, D. et al. Energy-adjusted ab initio pseudopotentials for second and third row transition elements. Theor. Chim. Acta 77, 123-141 (1990).

16 Klamt, A. & Schüürmann, G. COSMO: a new approach to dielectric screening in solvents with explicit expressions for the screening energy and its gradient. J. Chem. Soc., Perkin Trans. 2, 799-805, (1993).
